# The Delta variant wave in Tunisia: Genetic diversity, spatio-temporal distribution and evidence of the spread of a divergent AY.122 sub-lineage

**DOI:** 10.3389/fpubh.2022.990832

**Published:** 2023-01-04

**Authors:** Sondes Haddad-Boubaker, Marwa Arbi, Oussema Souiai, Anissa Chouikha, Wasfi Fares, Kate Edington, Sam Sims, Cesare Camma, Alessio Lorusso, Moussa Moïse Diagne, Amadou Diallo, Ilhem Boutiba Ben Boubaker, Sana Ferjani, Maha Mastouri, Salma Mhalla, Hela Karray, Saba Gargouri, Olfa Bahri, Abdelhalim Trabelsi, Ouafa Kallala, Naila Hannachi, Yassine Chaabouni, Hanen Smaoui, Khaoula Meftah, Sophia Besbes Bouhalila, Soumaya Foughali, Mariem Zribi, Asma Lamari, Henda Touzi, Mouna Safer, Nissaf Ben Alaya, Alia Ben Kahla, Mariem Gdoura, Henda Triki

**Affiliations:** ^1^Laboratory of Clinical Virology, WHO Regional Reference Laboratory for Poliomyelitis and Measles for the EMR, Institute Pasteur de Tunis, University of Tunis El-Manar, Tunis, Tunisia; ^2^Laboratory of Viruses, Hosts and Vectors, Institute Pasteur de Tunis, University of Tunis El Manar, Tunis, Tunisia; ^3^Clinical Investigation Center (CIC), Institute Pasteur de Tunis, University of Tunis El Manar, Tunis, Tunisia; ^4^Laboratory of Bioinformatics, Biomathematics and Biostatistics, Institute Pasteur de Tunis, University of Tunis El-Manar, Tunis, Tunisia; ^5^New Variant Assessment Platform (NVAP), UK Health Security Agency, London, United Kingdom; ^6^Department of Virology, Instituto Zooprofilattico Sperimentale dell'Abruzzo e del Molise G. Caporale (IZSAM), Teramo, Italy; ^7^Department of Virology, Pasteur Institute of Dakar, Dakar, Senegal; ^8^Laboratory of Microbiology, Charles Nicolle Hospital, Tunis, Tunisia; ^9^Laboratory Research Antimicrobial Resistance, Faculty of Medicine of Tunis, University of Tunis El-Manar, Tunis, Tunisia; ^10^Laboratory of Microbiology, Fattouma Bourguiba Hospital, Monastir, Tunisia; ^11^Laboratory Research Laboratoire des Maladies Transmissibles et Substances Biologiquement Actives, Faculty of Pharmacy of Monastir, University of Monastir, Monastir, Tunisia; ^12^Laboratory of Microbiology, Habib Bourguiba Hospital, Sfax, Tunisia; ^13^Laboratory of Microbiology and Biochemistry, Aziza Othmana Hospital, Tunis, Tunisia; ^14^Laboratory of Virology, Sahloul Hospital of Sousse, Sousse, Tunisia; ^15^Laboratory of Microbiology, Farhat Hached Hospital of Sousse, Sousse, Tunisia; ^16^Laboratory of Medical Biology, Ibn El Jazzar Hospital, Kairouan, Tunisia; ^17^Department of Microbiology, Faculty of Medicine of Tunis, University of Tunis El Manar, Tunis, Tunisia; ^18^Laboratory of Microbiology, Microbiology of Children and Immunocompromised, Faculty of Medicine of Tunis, University of Tunis El Manar, Tunis, Tunisia; ^19^Laboratory of Microbiology, Bechir Hamza Children's Hospital, Tunis, Tunisia; ^20^Laboratory of Medical Biology and Blood Bank, Institute Mohamed Kassab d'orthopédie, Manouba, Tunisia; ^21^Laboratory of Medical Biology, Menzel Bourguiba Hospital, Bizerte, Tunisia; ^22^Laboratory of Microbiology, La Rabta Hospital, Tunis, Tunisia; ^23^National Observatory of New and Emergent Diseases, Tunis, Tunisia; ^24^Department of Virology, Faculty of Pharmacy of Monastir, University of Monastir, Monastir, Tunisia

**Keywords:** SARS-CoV2, Delta variant, AY.122, phylogeny, spatio-temporal dynamic, next-generation sequencing

## Abstract

**Introduction:**

The Delta variant posed an increased risk to global public health and rapidly replaced the pre-existent variants worldwide. In this study, the genetic diversity and the spatio-temporal dynamics of 662 SARS-CoV2 genomes obtained during the Delta wave across Tunisia were investigated.

**Methods:**

Viral whole genome and partial S-segment sequencing was performed using Illumina and Sanger platforms, respectively and lineage assignemnt was assessed using Pangolin version 1.2.4 and scorpio version 3.4.X. Phylogenetic and phylogeographic analyses were achieved using IQ-Tree and Beast programs.

**Results:**

The age distribution of the infected cases showed a large peak between 25 to 50 years. Twelve Delta sub-lineages were detected nation-wide with AY.122 being the predominant variant representing 94.6% of sequences. AY.122 sequences were highly related and shared the amino-acid change ORF1a:A498V, the synonymous mutations 2746T>C, 3037C>T, 8986C>T, 11332A>G in ORF1a and 23683C>T in the S gene with respect to the Wuhan reference genome (NC_045512.2). Spatio-temporal analysis indicates that the larger cities of Nabeul, Tunis and Kairouan constituted epicenters for the AY.122 sub-lineage and subsequent dispersion to the rest of the country.

**Discussion:**

This study adds more knowledge about the Delta variant and sub-variants distribution worldwide by documenting genomic and epidemiological data from Tunisia, a North African region. Such results may be helpful to the understanding of future COVID-19 waves and variants.

## Introduction

Since December 2019, Severe Acute Respiratory Syndrome Coronavirus 2 (SARS-CoV2) emerged and spread rapidly all around the world, causing a global health, social and economic crises (1–3). The World Health Organization (WHO), all international expert networks and national public health authority institutions have been mobilized to monitor the evolution of SARS-CoV-2 and track emergent variants and sub-variants. Variant surveillance is based on genetic characterization and rapid sharing of viral genomic sequences, in order to limit their spread and control the pandemic (4). SARS-CoV-2 variants are classified as Variants Of Concern (VOCs), Variants Of Interest (VOIs) and Variants Under Monitoring (VUMs) (5). VOCs are associated to increased transmissibility and/or virulence, and/or to reduced efficacy of diagnostic tests, vaccines or treatments. VOIs are associated with significant community transmission with multiple foci in multiple countries and/or increasing prevalence. VUMs present unclear phenotypic or epidemiological repercussions requiring enhanced surveillance.

Among VOCs, the Delta variant posed an increased risk to the global public health and rapidly displaced the then dominant Alpha variant globally and changed the epidemiological infection landscape of this virus during 2021 (6). Delta variant is the common name used to refer to the B.1.617.2 Pango-lineage (7). In late 2020/early 2021, the parental strain B.1.617 emerged and rapidly spread to at least 60 countries (8–10). The first detected sub-lineage was B.1.617.1, also named Kappa variant, followed by B.1.617.2, the Delta variant, and B.1.617.3, the Epsilon variant (7). The Delta variant is one of the most contagious variants of the SARS-CoV-2. First detected on March 01 2021, it spread rapidly to almost all countries of the world, replacing the Alpha variant which was predominating globally (7, 11). Delta harbors mutations along the genome; among those in the gene encoding the spike protein, the substitutions T19R and T478K as well as the deletion E156-F157 may enhance antibodies' evasion causing escape from the host immune response (11, 12) and L452R, P681R may help cell entry by enforcing attachment to the ACE2 receptor and membrane fusion (13–15). Several studies demonstrated increased replication fitness (6) and reduced sensitivity to neutralizing antibodies from past infection (14) which contributed to the deadly epidemic wave in India during the first quarter of 2021, as well as the epidemic waves in England, South Africa and then almost all countries in the world (16–20). Up to February 2022, Delta continued to circulate globally, even after the emergence and spread of Omicron variant, infecting especially unvaccinated populations or in-complete vaccinated populations, including children. Furthermore, an important variability of this variant was reported in different areas of the world. Up to 2022-11-03, Delta variant has been shown to circulate into 245 different sub-lineages: AY.1-AY.134 (8).

At the beginning of COVID-19 pandemic, Tunisia adopted a very stringent lockdown regime from 10th of March to 3rd of May 2020 which helped contain the virus spread. Since July 2020, the country experienced several waves of COVID-19. The first two ones, in 2020 and up to January 2021, were caused by several SARS-CoV2 lineages (21–23). The first detected VOC was the Alpha variant that induced an extensive wave from March to June 2021. In May 2021, Delta was detected for the first time and started replacing the Alpha variant, causing a substantial increase in the number of cases with high transmission rates within the community (23). It became rapidly the dominant variant in the country with great impact on public health and healthcare systems, increasing the number of hospitlaisation requirement for oxygen. By the end of December 2021, a slight decline of COVID-19 incidence was notified but, since January 2022, the number of cases increased again causing another wave mainly induced by the Omicron variant.

This study reported demographic and genetic data of 662 SARS-CoV2 Tunisian sequences belonging to the Delta variant, obtained from oropharyngeal swabs collected from May to December 2021, during the Delta wave. The genetic diversity and the spatio-temporal dynamics of virus transmission in the country are described.

## Materials and methods

### Ethical statement

This study was performed under ethical standards according to the 1964 Declaration of Helsinki and its later amendments. The samples were collected in the context of COVID-19 diagnostic activities. They were used in the present study after de-identification with respect to patient anonymity and after the approval of the Bio-Medical Ethics Committee of Pasteur Institute of Tunis, Tunisia (2020/14/I/LR16IPT/V1). Written informed consent to participate in this study was provided by the participants ‘legal guardian. No animal studies are presented in this manuscript. No potentially identifiable human images or data is presented in this study.

### Tunisian sequences

**Figure 1:**
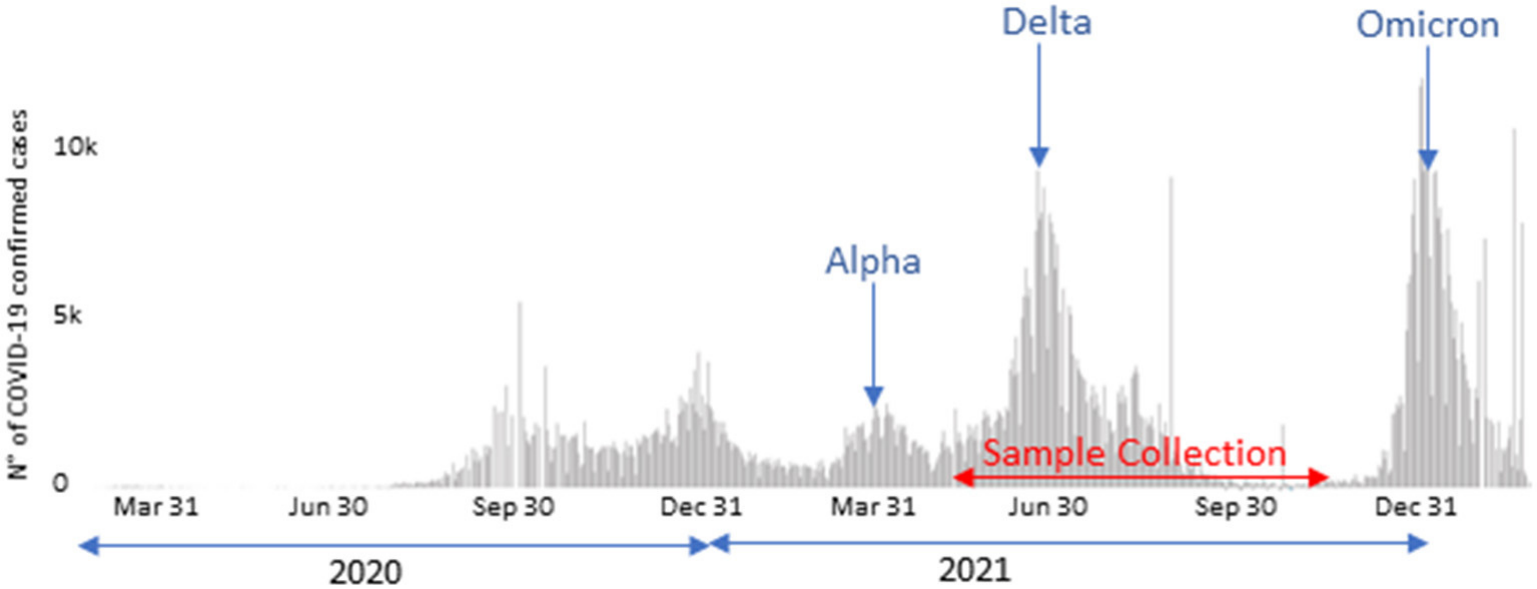
COVID-19 confirmed case counts in Tunisia starting from March 2020 (https://covid19.who.int/region/emro/country/tn, accessed by 18/03/2022) and sample collection period.

A total of 662 SARS-CoV-2 sequence, obtained from positive nasopharyngeal samples and lately identified as belonging to the Delta variant, were included in the present work. They were obtained from May, 26 to December 1st, 2021, during the Delta wave in Tunisia (Figure 1). The samples were collected by the Tunisian Ministry of Health personnel (MoH's personnel). They included samples collected from home-quarantined and hospitalized individuals. The collected samples were transported, under refrigeration and within 24 h, to the Pasteur Institute of Tunis where they were immediately processed for SARS-CoV-2 RNA detection by specific real-time reverse transcription PCRs (RT-qPCR) according to WHO-approved protocols (24, 25). Samples were tested by Charité, Berlin protocol [singleplex envelop (E) and singleplex RNA-dependent RNA polymerase (RdRp)] (24), Hong Kong Universiy, China protocol (singleplex nucleoprotein (N) and singleplex Open reading frame Orf1b) (25), according to the reagents availability, in the pandemic context. SARS-CoV-2 variant identification was assessed using a combination of next-generation sequencing-based approaches (*N* = 484 samples) and by partial sequencing of the S gene (*N* = 178 samples).

### PCR amplification and partial sequencing in the S gene

PCR amplification of S gene was performed as previously described (22) with minor modifications, using new designed primer's pair RBF_FW & IPT_REV, that allowed the amplification of a 1,112-nucleotides segment. Briefly, after viral RNA extraction using the QIAamp MinElute Virus Spin Kit (Qiagen, GmbH, Hilden, Germany), the amplification used the primer's pair RBF_FW: 5'*ATTACAAACTTGTGCCCTTTT*3' (positions 22535 to 22555 according to the SARS-CoV2 reference genome, NC_045512.2) and IPT_REV: 5′*CTGCACCAAGTGACATAGTG*3′ (positions 23666 to 23647) and the SuperScript III one-step RT-PCR system with the Platinum *Taq* DNA polymerase kit (Invitrogen). The S amplicons were purified using the ExoSAP-IT method (Invitrogen). Sequences were generated by mean of Big Dye terminator chemistry according to the manufacturer's recommendations (BigDye Terminator v3.1 kit, Applied Biosystems) and using the automated sequencer ABI 3130). The consensus sequences of approximately 1,071 nucleotides were deduced using Clustal X version 2.0 (http://www.clustal.org/) (26).

### Whole genome sequencing

The sequencing of complete SARS-CoV2 viral genome was achieved using NGS technologies in 3 collaborating institutes: 396 at the Quadram Institute (Norwich, United Kingdom) on behalf of the UKHSA New Variant Assessment Platform, 50 at the Istituto Zooprofilattico Sperimentale dell'Abruzzo e del Molise (Teramo, Italy) (IZS-Te) and 38 at Institut Pasteur de Dakar (Senegal). The three sequencing sites used almost equivalent sequencing protocols as previously described (22). RNA was extracted from a 140 μL nasopharyngeal sample with the Qiamp viral RNA mini kit (Qiagen, Hilden, Germany) according to the manufacturer's instructions. The extracted RNAs were reverse transcribed to single-strand cDNA. The whole genome sequencing was assessed using the Illumina COVIDSeq Test, including reagents for cDNA amplification (CPP1 HT and the CCP2 HT primers), library preparation and Purification (Illumina Tune Bead) (Illumina Technology, USA). The quantification of amplification products was assessed by the Qubit 2.0 fluorometer (ThermoFisher Scientific, USA) and qualified on an Agilent Technologies 2100 Bioanalyser using a high-sensitivity DNA chip following the manufacturer's instructions. Generated libraries were pooled, denatured, diluted to 1.4 pM and sequenced using NextSeq 500/550 High output Kit version 2.5 (Illumina, Inc, USA) on a NextSeq500 or NextSeq550 instrument (Illumina, Inc, USA) providing 2 × 150 bp read length data.

The quality control of raw data was first checked using FastQC version 0.11.9 for (https://www.bioinformatics.babraham.ac.uk/projects/fastqc/). Low-quality reads and adapters were filtered using Trimmomatic version 0.39 (27) with a Phred quality score of 30 as the threshold. Consensus sequences were generated by mapping to the SARS-CoV-2 reference genome (NC_045512) *via* Spades assembler version 3.15.0 (28), using thresholds of 80% for nucleotide sequence coverage and 90% for nucleotide similarity. The sequences were submitted to the GISAID database (https://www.gisaid.org) (29, 30).

### Variant assignment and single nucleotide polymorphisms identification

The Fasta files of the whole sequences were uploaded into Pangolin online software version 3.1.17 (https://cov-lineages.org/pangolin.html; accessed on 01/16/2022) for variant and sub-lineage assignment (31). Sub-lineages within the Delta variant could be assigned for those having a coverage of more than 85% of the virus genome. The web-based software Nextclade v1.12.0 (https://clades.nextstrain.org/) was used for clade assignment, diversity and gene-wise amino acid mutations analysis (32). Maximum-likelihood trees were generated by the use of IQ-TREE multicore program version 1.6.12 (33).

For the 178 partial sequences and the NGS sequences with incomplete coverage of the whole genome, variant determination was based on the identification of key mutations specific to the Delta variant along the partial and complete S gene, respectively.

### Mutational cascade analysis

Mutational cascade was analyzed for AY.122 variant of SARS-CoV-2 circulating in Tunisia using NUCmer pipeline (version 4.0.0) and the R script detailed by Bansal and Kumar (34). The SNP analysis was conducted by NUCmer v.4.0.0 using genome of first reported AY.122 variant (OV103802.1). This reference genome was retrieved from NCBI Genbank (https://www.ncbi.nlm.nih.gov/nuccore/OV103802.1/) and chosen for no N character in the nucleotide sequence as selection criteria. The SNP file generated by NUCmer v.4.0.0, the R script was launched to identify: most mutated samples, number of mutations per sample, mutation classes, and most frequent mutational events per type. In this analysis, a gff3 annotation file of the Wuhan reference genome (NC_045512.2) was used to extract coordinates of the proteins encoded by SARS-CoV-2 genome.

### Phylogenetic analysis

Viral phylogeny was inferred on Tunisian generated sequences and others originating from different areas of the world, available at the NCBI GenBank (https://www.ncbi.nlm.nih.gov/labs/virus). Multiple alignment was first inferred by Mafft (V7.470) online platform (https://mafft.cbrc.jp/alignment/software/), using default parameters (35). A Maximum-likelihood tree was generated by IQ-TREE multicore program version 1.6.12, with a bootstrap replication of 1000 cycles (33, 36). The Wuhan reference genome (NC_045512.2) was used for rooting. The visualization of the obtained tree was assessed by the graphical viewer program, FigTree v1.4.4 (http://tree.bio.ed.ac.uk/) (37).

### Phylogeographic analysis

To explore spatio-temporal distribution of investigated strains, the temporal signal was undertaken using TempEST v1.5.4 (38). Bayesian phylogeographic analysis used BEAST v1.8.4 (39), the discrete diffusion model approach was applied (40). The GTR (I+Γ4), strict clock and exponential growth models are applied as the best-fit models. To estimate the transition rates across regions, the Bayesian Stochastic Search Variable Selection (BSSVS) approach was inferred with application of the symmetric substitution model. A 200-million iteration was run, achieving a satisfying ESS value (ESS>200). By discarding 10% of burn-in, the time-scaled maximum clade credibility (MCC) tree was generated with Tree Annotator v1.8.4 (40) and visualized with FigTree v1.4.3 (37) programs. To evaluate the robustness of virus transitions between geographic locations, the Bayes factors (BF) were calculated by using the SpreaD3 program version 1.0.7 (41). A transition rate is considered as statistically valid when BF>3. The spatiotemporal dynamics of strains was studied by generating a KML file with the SpreaD3 program and then visualized *via* Google Earth Pro.

### Data availability

Complete genome SARS-CoV-2 sequences generated in this study were submitted to the GISAID database (https://www.gisaid.org) (29, 30). Their accession numbers are provided in Supplementary Table S1.

## Results

### Epidemiological features of collected samples

**Figure 2:**
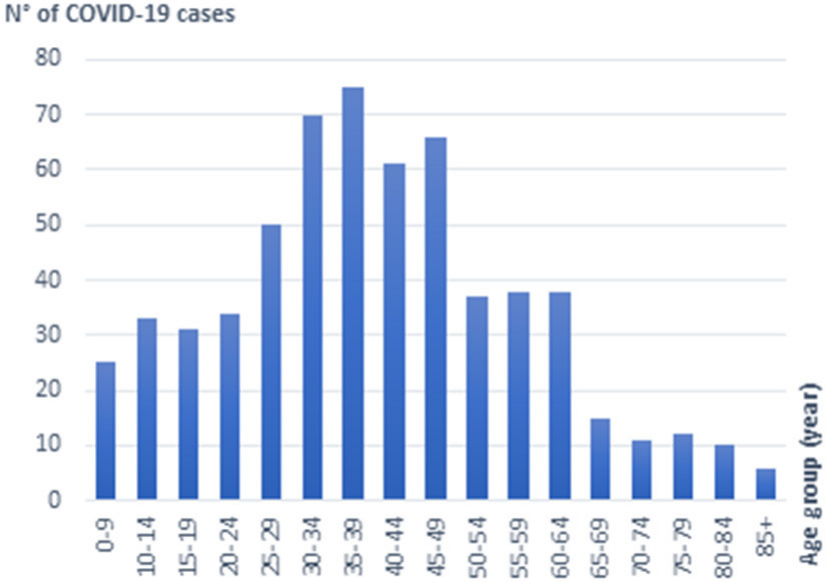
Distribution of Delta SARS-CoV2 sequences according to the age.

The Delta sequences included in this study were obtained from individuals originating from 23 of the 24 Tunisian governorates. They were collected from symptomatic patients with a wide range of clinical presentations, as well as asymptomatic individuals sampled after contact with confirmed cases. The study population included 303 males and 359 females; their ages ranged from 1 month to 90 years. Figure 2 displays the distribution of SARS-CoV2 sequences according to the age. More than half of the sequences (52.6%) were obtained from individuals aged 25 to 50 years, with statistical significant difference (*P* = 0.0004 <0.001), obtained by student's *t*-test. An important proportion is also found among children and teenagers (20.0%) as well as seniors aged 50–65 (18.4%). Only 9.0% of sequences were obtained from individuals aged 65 years and more.

### Sequence analysis and variant assignement

Among the 484 whole genome sequences, variant and sub-variant could be assigned for 468 sequences displaying <15% ambiguous nucleotides.

Using Nextclade program, the 468 complete sequences belonged to clades 21J, 21I and 21A which correspond to the Delta variant. The majority of sequences constitute a group belonging to the 21J clade and other sporadic strains grouped within the 21A, 21I clades (Supplementary Figure S1). Similar group profile was obtained with the Maximum Likelihood (ML) tree obtained by Iqtree software (Supplementary Figure S2).

**Figure 3:**
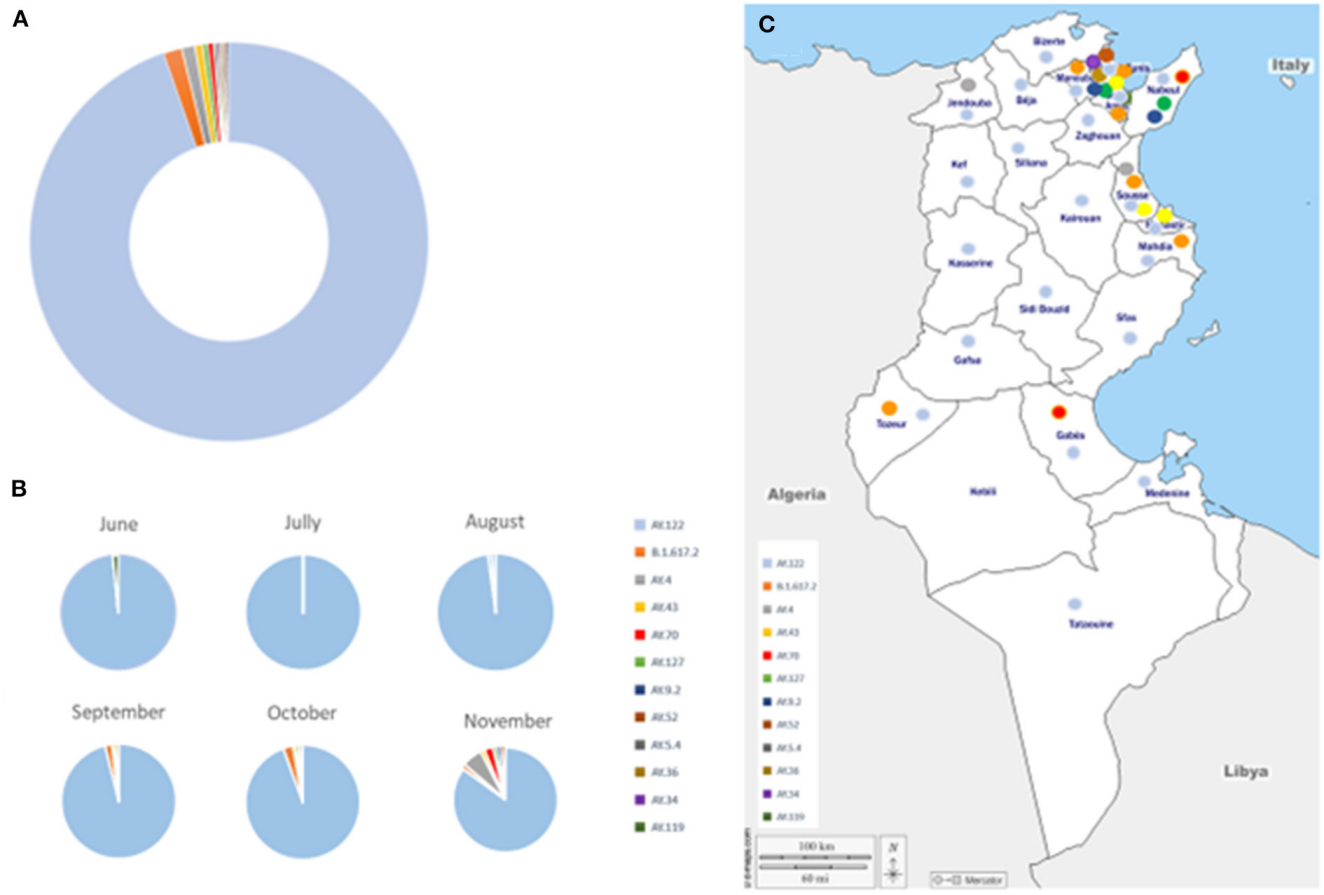
Distribution of Delta sub-lineages, during the study period. **(A)** Global distribution. **(B)** Temporal distribution. **(C)** Geographic distribution of Delta sub-lineages, during the study period. Delta sub-lineages are indicated by colored circles, as indicated in the legend.

Using Pangolin program, the 468 analyzed sequences were classified into 12 different Delta sub-lineages: AY.122, B.1.617.2, AY.4, AY.43, AY.70, AY.127, AY.9.2, AY.52, AY.5.4, AY.36, AY.34, AY.119. The AY.122 sub-lineage was the most frequently detected (94.6%, *n* = 443) followed by the B1.617.2 (1.4%, *n* = 7) and the AY.4 (1.0%, *n* = 5) sub-lineages (Figure 3A). For the other minor sub-lineages, the number of sequences ranged from 1 to 3 each.

The AY.122 sub-lineage was detected since May and circulated during the whole study period, up to December 2021 while the frequency of detection of the other sub-lineages increased since August 2021 and was the highest in November 2021 (Figure 3B). Four, five and eight different sub-lineages were detected in September, October and November 2021, respectively. AY.122 was detected in all regions of the country while the other sub-lineages were mainly detected in the North-east-coastal regions (Figure 3C).

### Mutational cascade

**Figure 4:**
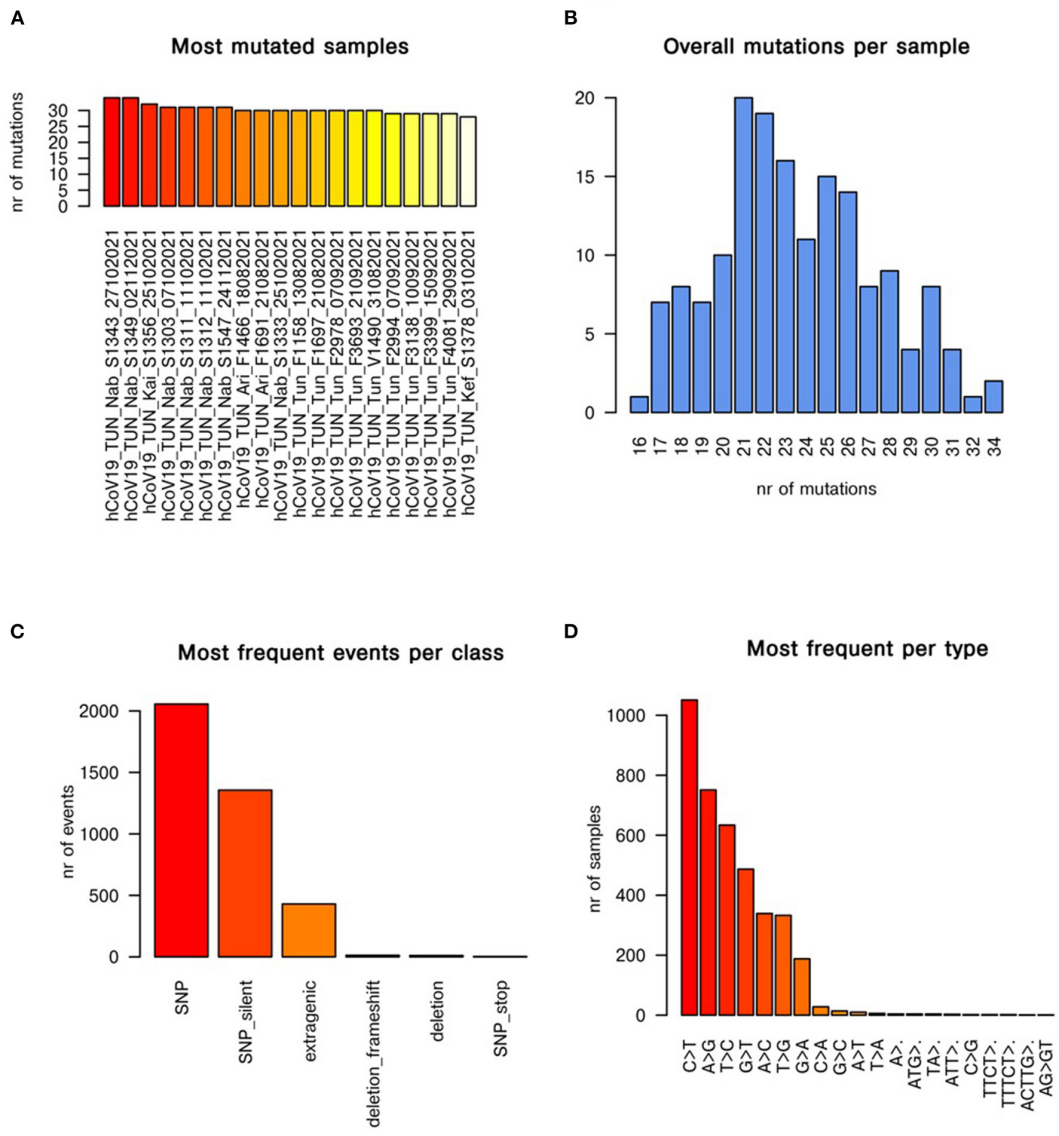
Mutational cascade of AY.122 variant emerged in Tunisia with respect to first reported AY.122 strain. The panels **(A–D)** show the most mutated samples, mutations per sample, most frequent events per class of mutation, most frequent mutational events per type, respectively.

For more investigation about evolution trait of emergent AY.122 variant (*n* = 165) in Tunisia, we constructed the mutational cascade based on comparison with the first reported AY.122 strain (Figure 4). According to the mutational analysis, the most mutant strains are: hCoV-19_TUN_Nab_S1343_27102021 and hCoV-19_TUN_Nab_S1349_02112021, where they accumulated more than 30 mutations in their genomes (Figure 4A). Figure 4B showed that most genomes accumulated 20 to 26 mutations during AY.122 variant emergence in Tunisia. According to the Figure 4C, the single nucleotide polymorphism (SNP) is the most prevalent mutation class among the AY.122 variant circulating in Tunisia. The mutational analysis also showed that C>T and A>G transitions are the most frequent mutation events (Figure 4D).

### Phylogenetic analysis

The phylogenetic analysis was performed on the AY.122 complete genome sequences, representing the most frequently detected Delta sub-lineage. The analysis involved a total of 391 AY.122 virus genomes including the Wuhan reference sequence as outgroup, 165 Tunisian sequences with complete genome coverage and 225 sequences from different geographic regions of the world. Indeed, to obtain reliable results, only complete sequences without ambiguous nucleotides (N) into the coding regions were considered. The 165 AY.122 complete genome Tunisian sequences originated from 17 different governorates and during the whole study period. Among the AY.122 complete genome sequences from other countries, available on Genbank, up to10 complete sequences per country and per month were first selected (*n* = 780) and then only those with without ambiguous nucleotides into the coding regions (*n* = 225) were retained. They included sequences for United Kingdom (*n* = 43), Germany (*n* = 38), United States of America (*n* = 29), India (*n* = 24), Egypt (*n* = 18), Bahrain (*n* = 14), Slovakia (*n* = 11), Italy (*n* = 10), Nigeria (*n* = 10), China (*n* = 8), Austria (*n* = 6), France (*n* = 6), Liechtenstein (*n* = 4), Mongolia (*n* = 2), Russia (*n* = 1).

**Figure 5:**
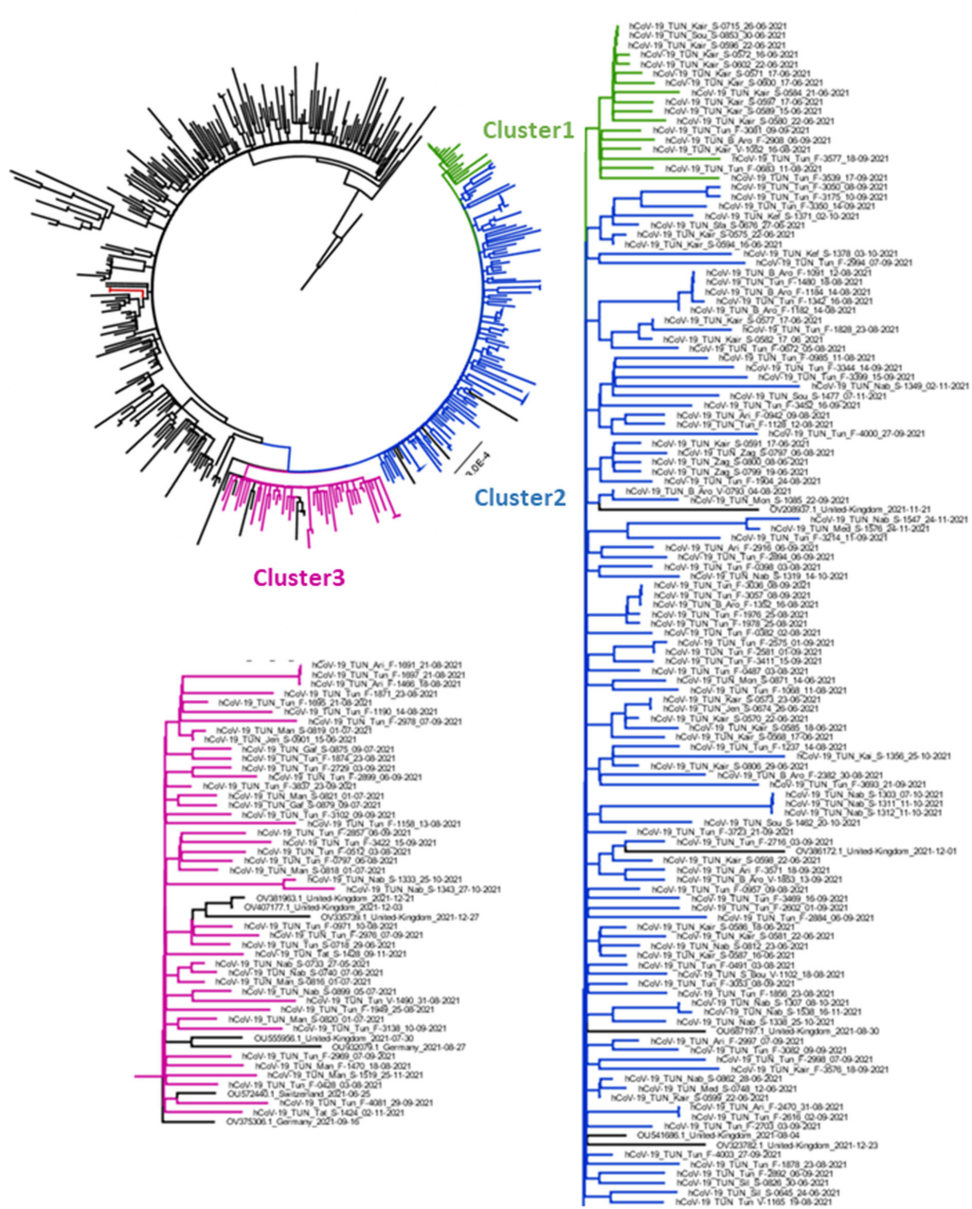
Phylogenetic analysis of AY.122 sub-lineage's Delta strains obtained in Tunisia between May and December. The Maximum Likelihood tree was obtained by comparison of 391 High quality AY.122 genomes obtained from Tunisia and other regions of the word. Tunisian sequences are indicated in Green (Cluster1), Blue (Cluster 2) and Pink (Cluster3) and other sequences different the world in Black. The tree was generated by IqTree software with 1000 boostrap cycles and visualized by FigTree software.

Figure 5 represents the Maximum-Likelihood tree generated using the GTR+F+R2 model (Best fit model according to BIC) with a bootstrap replication of 1000 cycles. The majority of Tunisian sequences (163 out of 165) clustered together, independently from sequences of other countries. They were grouped into three major clusters. Cluster1 included 17 Tunisian sequences collected in limited areas of Tunisia (Tunis and Kairouan); they formed two groups according to their geographic origin. Cluster2 included a large number of Tunisian sequences (*n* = 105) from different governorates. Sequences from same region were highly related and likely to form different groups. This cluster included few sequences from UK. The Cluster 3 combines 43 sequences from three Tunisian regions (the region of Tunis, Nabeul and South Tunisia) with few sequences from Germany, Switzerland and UK.

**Figure 6:**
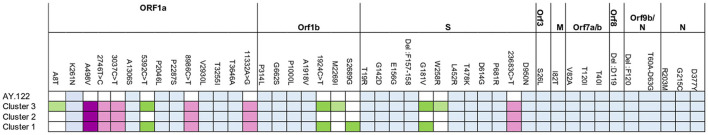
Mutational profile of Tunisian sequences from Cluster 1, 2 and 3 in comparison with the AY.122 sub-lineage.

Figure 6 displays the mutational profile of the Tunisian sequences. The web-based software Nextclade v1.12.0 (https://clades.nextstrain.org/) was used for gene-wise amino acid mutations analysis (32). In addition to the amino-acid changes and mutations specific to the AY.122 sub-lineage, indicated in blue, all Tunisian sequences, from the three clusters, shared the amino-acid change A498V in ORF1a (indicated in dark purple), the synonymous mutations 2746T>C, 3037C>T, 8986C>T and 11332A>G in ORF1a and 23683C>T in the S gene (indicated in light purple). The same changes were also found with high frequency in all the 443 Tunisian AY.122 sequences: A498V was present in 441 sequences, 2746T>C, 3037C>T, 8986C>T, 11332A>G were found in 418, 441, 437 and 439 sequences, respectively and 23683C>T was found in 433AY.122 sequences.

In addition, the Tunisian sequences from Clusters 1 and 3 differ from the cluster 2 by the presence of the amino-acid changes S: G181V and the synonymous mutations 5392C>T, 19245C>T (indicated in Green). Also, strains from Cluster 3differ from the other by the presence of ORF1b: S2689G amino acid change.

Along the genome of Tunisian sequences, other amino-acid changes were present at important levels in the S gene: G142D (121/165 sequences), S: W258R (33/43 sequences from the Cluster3) and ORF1b gene M2269I (9/43sequences from the Cluster 3) and also ORF7a gene A8T (5/43 sequences from Cluster 3).

### Spatio-temporal dynamics of AY.122 Tunisian sequences

From the data set composed of the 165 AY.122 Tunisian sequences, with no ambiguous nucleotides, an ML tree was constructed and used to explore the temporal signal of the selected dataset. The root-to-tip regression analysis shows a positive correlation (R^2^ = 0.37) between genetic distances and sampling dates (Supplementary Figure S3).

Molecular clock analysis shows that the TMRCA (Time to the Most Recent Common Ancestor) for the root of AY.122 variant in Tunisia was estimated to be around October 03th, 2020 [July 19th, 2020–January 02th, 2021], which could correspond to the date of its first emergence in the world (6).

To gain insight into spatio-temporal dynamics of the AY.122 variant dispersal in Tunisia, we performed a Bayesian analysis based on phylogeography framework reconstruction. In the time-scaled phylogeographic MCC (Maximum Clade Credibility) tree, we observed that most nodes and branches have colors assigning the inferred location of Tunis (Supplementary Figure S4). Furthermore, the root state posterior probability (RSPP) analysis shows that Tunis has the highest value (RSPP = 0.95). This finding indicates that Tunis might be responsible for the spread of the AY.122 Delta SARS-CoV-2 variant in the country.

**Figure 7:**
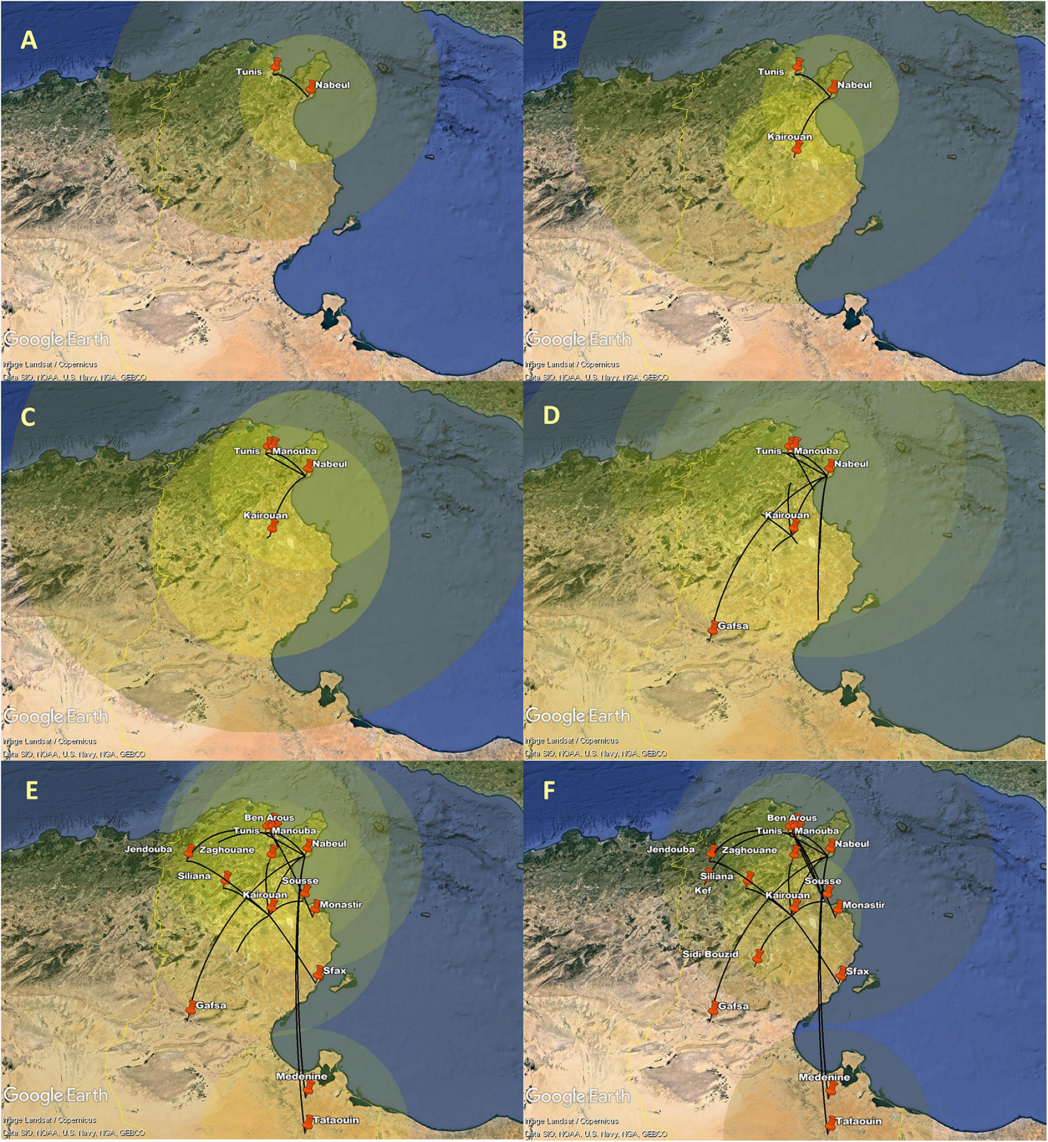
Spatial dynamics of AY.122 Delta variant of SARS-CoV-2 among different Tunisian governorates. The snapshots **(A–F)** show the different stages of AY.122 variant spread in Tunisian governorates. Transition lines linking different locations represent the branches in the MCC. Diameters of the circles are proportional to square root of the number of MCC branches maintaining a particular location state at each time point.

The spatial estimates of the MCC tree were than mapped on Google Earth (Figure 7, Supplementary material 1). The generated configuration of spatial dynamics shows that the AY.122 dispersal in Tunisia started from Tunis (the capital) to Nabeul (North-east). The virus was transmitted then from Nabeul to Kairouan, in the center of the country. Soon after, the role of Nabeul as primary epicenter of AY.122 variant became clearer; transitions were observed transitions with Manouba (North-east) and then with Gafsa (central-west). In this period, the AY.122 infection zones started to expand across the country from Nabeul, Tunis and Kairouan. Subsequently, Tunis and Kairouan became secondary epicenters of intensified AY.122 outbreaks which affected more governorates in the North, in the Center, and the South.

**Figure 8:**
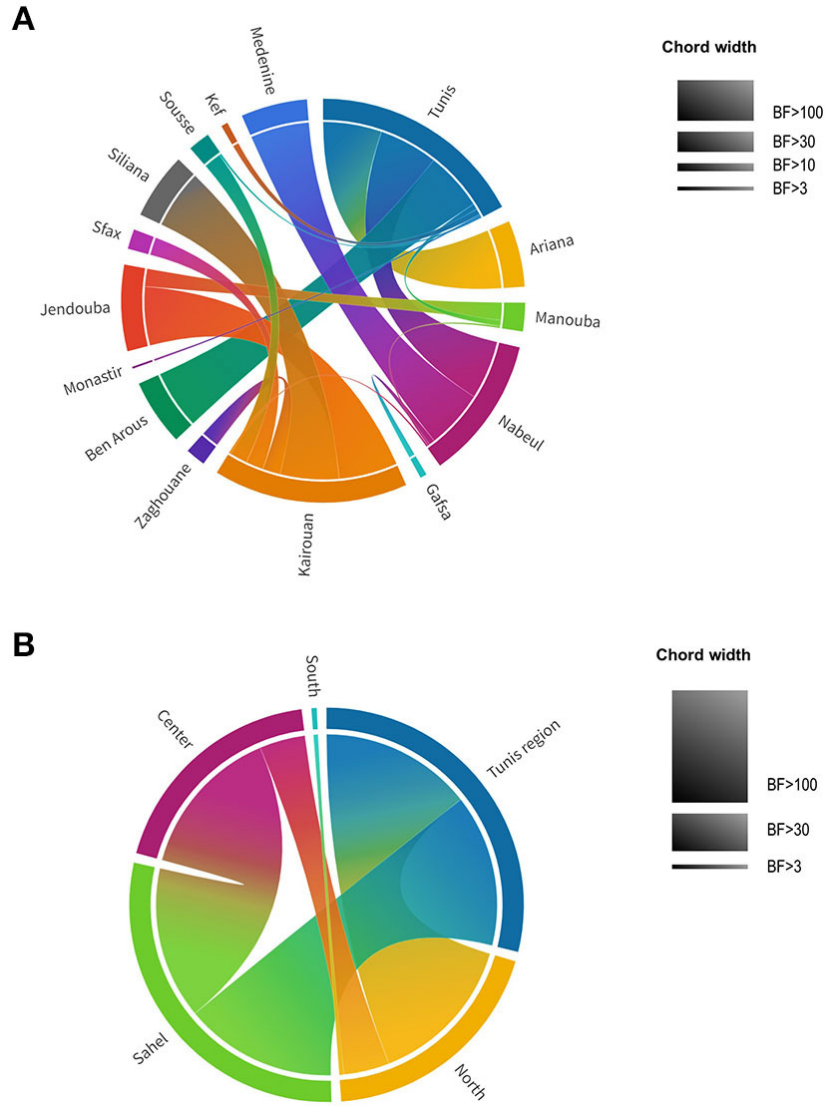
Chord diagram of theAY.122 SARS-CoV-2 variant representing Bayes Factor (BF) of transitions between different locations in Tunisia. **(A)** BF of transitions between Tunisian governorates. **(B)** BF of transitions between Tunisian regions. Tunisian governorates and regions are presented by different colors. Chord width is proportional to BF support levels: BF <3 no support, BF > 3 substantial support, BF > 10 strong support, BF > 30 very strong support, BF > 100 decisive support.

To provide statistical support for the phylodynamic reconstruction, we determined the Bayes Factor (BF) for the identified transitions between the different Tunisian governorates (Figure 8A, Supplementary Table S2). We found that all the transitions between the governorates are statistically supported (BF>3) except for the transition “Nabeul–Tataouine” (BF = 2.58). The highest Bayes Factor (BF>100) was recorded for the transitions linking: Tunis to Ariana (BF = 137653.83), Tunis to Nabeul (BF = 12132.57), Tunis to Ben Arous (BF = 1376535.83), Nabeul to Medenine (BF = 259.69), Kairouan to Siliana (BF = 201.58) and Kairouan to Jendouba (BF = 117.36).

Higher decisive support of the Bayes Factor was found when governorates were assembled by regions (Figure 8B, Supplementary Table S3): Tunis region (Tunis, Manouba, Ariana, Ben Arous), North (Bizerte, Nabeul, Jendouba, Béjà, Zaghouan), Sahel (Sousse, Monastir, Mahdia, Sfax), Center (Siliana, Sidi Bouzid, Kasserine, Kairouan), South (Tataouin, Mednine, Kebili, Gafsa, Tozeur). For the region trait, all the Bayes Factors were superior to 3 indicating the statistical validation for the identified transitions. The transitions having a decisive support of Bayes Factor (BF > 100) are those of: “Tunis region–North” (BF = 294254.38), “Tunis region–Sahel” (BF = 104.44) and “Center–Sahel” (BF = 269.06).

## Discussion

Since May 2021, the Delta variant dominated all the other SARS-CoV-2 variants and continued to largely shape the pandemic up to February 2022 (15). It overwhelmed health care systems, infecting mainly unvaccinated and non-fully vaccinated populations, especially in countries with limited access to vaccines (41, 42).

The Delta wave started in Tunisia, in May 2021. The present work shows that Delta infections occurred mainly in young adults. The age distribution of investigated cases showed a large peak between the ages of 25 to 50 years, but several cases were also found in children and teenagers, as well as seniors aged 50 to 65 years. Indeed, COVID-19 vaccination started in Tunisia only by March 13, 2021, with a slow cadence. Health worker and individuals aged 75years and over were first vaccinated, followed by individuals from age groups 65–74 and then 60–64 years (43). Low vaccination coverage was achieved by May 2021, when Delta was introduced, and slow progression of vaccination campaigns was reported. Vaccination of individuals from age groups 50–59, 40–49, 30–39 was initiated progressively from June 1, to August 9, 2021(44–46). Vaccination of the age group 18–29 started later, by October 10, 2021 (47). Thus, the stuttering vaccination campaign, coupled the advent of the delta variant may be the cause of the deterioration of the health situation in Tunisia and explain the age distribution of Delta cases that we report herein. Furthermore, when Delta was introduced in the country, most of the already vaccinated peoples received only one dose which has been reported to be insufficient to induce effective protection against the Delta variant. Indeed, clinical evidence indicated decline in vaccine's effectiveness in the case of Delta variant infection (48). *In vitro* tests also showed lower neutralization activity of vaccine-induced antibodies against the Delta variant (49), as compared with the previous variants. Only administration of two COVID-19 vaccine doses may provide effective protection against the Delta variant (42, 48, 50, 51).

Worldwide, The Delta variant was characterized by a huge genetic variability, including the largest number of sub-lineages among VOCs. As of 2022-03-26, 234 different sub-lineages were reported (AY.1 to AY.133), represented by 4 259 793 sequences shared *via* GISAID, up (7). AY.4 is the most predominant sub-lineage in the world (19.5% of available Delta sequences), followed by AY.43, AY.103, AY.44; AY.122 comes in the sixth position (7). In our series and among all the Delta sub-lineages reported globally, twelve were detected in Tunisia with a large predominance of the AY.122 sub-lineage, which represented more than 94% of detected sequences. This sub-lineage was present in the country, from the beginning of the delta wave in May 2021 till its end in December 2021. It also spread to almost all regions of the country, including rural and urban regions. This suggests that it has established an autochthone transmission. Although, the number of samples obtained from rural regions is limited, the localization of main Tunisian population into the coastal regions around the big tows and the high connection between population in rural and urban regions are online with our finding. In the other hand, the eleven other variants were detected during shorter periods of time and in more restricted geographical areas, especially in the North-East region where are found the biggest cities and the main airports. This suggests that they were introduced through multiple importation events, mainly since August 2021, and that these minor sub-lineages were not able to establish autochthone transmission in the presence of the already circulating AY.122 sub-lineage. The restricted number of circulating sub-lineages during the Delta wave in Tunisia may also reflect the efficiency of public health measures taken place at the national gateways and entry points connected to the Tunisian border. The observation of isolation measures by travelers after arrival at the national territory may have modulated dispersion of numerous sub-lineages. In other contexts, such as in India, multiple introductions at the same time resulted in a rapid community spread and a wide dispersion of sub-lineages among the population (52). In Tunisia, the main population is located at coastal regions with important exchanges between rural and urban populations.

The AY.122 sub-lineage was first detected in India, 2020-09-07 (8). It's mainly circulating in Eastern-European regions such as Belarus, Armania, Russia (>40% of sequenced samples) and is also frequently detected in other European countries such as Monaco (>40%) and in Asian regions such as Kazakhstan and China (41 and 27%, respectively) (7). AY.122 also represents 70% sequenced samples in Seychelles (7). Given the strategic localization of Tunisia in the middle of the Mediterranean Sea, the high trade exchange with Europe, the high number of tourists visiting Tunisia and also the localization of the Tunisian population mainly in coastal regions, AY.122 may have been imported from Europe, especially from Eastern-Europe region. Indeed, an increased number of tourists coming from this region during the last years was recorded. In the other hand, countries such as Monaco and Seychelles also constitute privileged destinations for Tunisian tourists (53). Spatio-temporal analyses conducted in the present work suggest the introduction of the AY.122 sub-lineage first in Tunis, the capital and then its propagation to two other big cities that constituted after-on, together with Tunis, epicenters for the propagation of the virus all over the country. The mutational cascade describing important evolution of virus obtained in November-December, from big Tunisian cities confirm also our finding. Similar widespread of the same sub-lineage in Russia was recently reported (54). In Tunisia, and especially in big cities, it is evident that the local commercial exchanges, regular market visits and families' celebrations, during the summer time may have accelerated community transmission. The low SARS-CoV2 vaccine coverage achieved during the Delta wave as well as the decline in prevention measures within the population may explain the establishment of autochthone transmission.

Noticeably, the AY.122 Tunisian investigated sequences showed were highly related to each other and constituted an individualized genetic group independently from most of the sequences from other countries included in the phylogenetic tree. The mutational profile reveals that most of the Tunisian sequences share the A498V amino acid change in the ORF1a and 5 other synonymous mutations: 2746T>C, 3037C>T, 8986C>T, 11332A>G in ORF1a and 23683C>T in the S gene. In addition, the amino acid changes S: G181V; W258R were found with important rates. To the best of our knowledge, this is the first report of such amino acid changes among the AY.122 sub-lineage. The substitution A498V in ORF1a was previously reported among Wuhan-like strains Malaysian sequences, obtained during the first and second SARS-CoV2 waves, January-February 2020 (55). The G181V was reported in the Brazilian P.1 variant, but its impact was not investigated (56). The W258R in the S gene was reported among COVID-19 family clusters, including severe cases (57). The spread of viral lineages may rely on different factors such as virus properties, host genetic and immunological background, demography and socio-cultural behavior of the population and, probably, the combination of different factors. The AY.122 Tunisian strains may owe their successful spread to the presence of particular amino-acid substitutions. Even no evidence of increased virulence or transmission related to these changes were reported, It will be relevant to investigate their impact on protein structure and function to better understand the AY.122 spread.

In conclusion, this study adds to the knowledge on the Delta variant and sub-variant distribution worldwide and documents genomic data from Tunisia, North Africa. The introduction of a restricted number of delta variants in Tunisia may reflect the efficiency of public health measures taken place at the national gateways and entry points connected to the Tunisian border. Nevertheless, the decline in prevention measures within the population may explain the establishment of autochthone transmission of AY.122 sublineage.

## Data availability statement

The datasets presented in this study can be found in online repositories. The names of the repository/repositories and accession number(s) can be found in the article/Supplementary material.

## Ethics statement

The studies involving human participants were reviewed and approved by Bio-Medical Ethics Committee of Pasteur Institute of Tunis, Tunisia (2020/14/I/LR16IPT/V1). Written informed consent to participate in this study was provided by the participants' legal guardian/next of kin.

## Author contributions

SH-B, OS, ABK, and HTr: conceptualization. SH-B, ABK, MA, OS, AC, WF, MG, ALa, and HTr: data curation. SH-B, MA, AC, WF, OS, KE, SS, CC, ALa, MD, AD, MG, HTo, and ALo: investigation. MM, HK, SG, OB, AT, OK, NH, YC, HS, KM, SBB, SF, MZ, MS, NA, IB, SF, and HTr: resources. HTr: funding acquisition. SH-B, OS, ABK, MG, and HTr: supervision. SH-B, ABK, OS, and HTr: validation. SH-B, MA, ABK, OS, and HTr: visualization and original draft preparation. SH-B, MA, and OS: writing. SH-B and HTr: writing—review and editing. All authors reviewed the manuscript and agreed to its submission to this journal.
