# Supplementary material for: The Delta variant wave in Tunisia: Genetic diversity, spatio-temporal distribution and evidence of the spread of a divergent AY.122 sub-lineage
Source: Front Public Health. 2023 Jan 4;10:990832. doi: 10.3389/fpubh.2022.990832 (PMC9846204; doi:10.3389/fpubh.2022.990832)
Supplement: Supplementary file 1 [file Data_Sheet_1.PDF]

The Delta variant wave in Tunisia: Genetic diversity, spatio-temporal distribution and evidence of the spread of a divergent AY.122 sub-lineage

Sondes Haddad-Boubaker[1,2,3*], Marwa Arbi†[1,4], Oussema Souiai†[4], Anissa Chouikha[1,2,3], Wasfi Fares[1,2,3], Maha Mastouri[5], Hela Karray[6], Olfa Bahri[7], Halim Trabelsi[8], Naila Hannachi[9], Yassine Chaabouni[10],Hanène Smaoui[11, 12], Sophia Besbes Bouhalila[13], Soumaya Foughali[14], Mariem Zribi[15], Mariem Gdoura[1,2,3,16], Asma Lamari[1,2], Henda Touzi[1,2,3], Mouna Safer[17], Nissaf Ben Alaya[17], Alia Ben Kahla[4],IlhemBoutiba Ben Boubaker[18, 19], Henda Triki[1,2,3].

Supplementary Table1. Accession number of the 484 complete genome Tunisian investigated sequences

Virus name	Accession ID
hCoV-19/Tunisia/A-0077/2021	EPI_ISL_11266035
hCoV-19/Tunisia/A-0208/2021	EPI_ISL_8298444
hCoV-19/Tunisia/A-0209/2021	EPI_ISL_8298445
hCoV-19/Tunisia/E-1320/2021	EPI_ISL_10141394
hCoV-19/Tunisia/E-1332/2021	EPI_ISL_10141451
hCoV-19/Tunisia/E-1601/2021	EPI_ISL_10141430
hCoV-19/Tunisia/E-1609/2021	EPI_ISL_10141553
hCoV-19/Tunisia/E-1617/2021	EPI_ISL_10141414
hCoV-19/Tunisia/E-1634/2021	EPI_ISL_10141450
hCoV-19/Tunisia/E-1663/2021	EPI_ISL_10141440
hCoV-19/Tunisia/E-1674/2021	EPI_ISL_10141550
hCoV-19/Tunisia/E-1712/2021	EPI_ISL_10141544
hCoV-19/Tunisia/E-1720/2021	EPI_ISL_10141552
hCoV-19/Tunisia/E-1725/2021	EPI_ISL_10141463
hCoV-19/Tunisia/E-1738/2021	EPI_ISL_10141542
hCoV-19/Tunisia/E-1757/2021	EPI_ISL_10141449
hCoV-19/Tunisia/E-4801/2021	EPI_ISL_10141413
hCoV-19/Tunisia/E-5228/2021	EPI_ISL_10141391
hCoV-19/Tunisia/E-0527/2021	EPI_ISL_10141504
hCoV-19/Tunisia/F-0381/2021	EPI_ISL_8298386
hCoV-19/Tunisia/F-0382/2021	EPI_ISL_8298446
hCoV-19/Tunisia/F-0398/2021	EPI_ISL_8298447
hCoV-19/Tunisia/F-0407/2021	EPI_ISL_8298387
hCoV-19/Tunisia/F-0422/2021	EPI_ISL_8298388
hCoV-19/Tunisia/F-0428/2021	EPI_ISL_8298448
hCoV-19/Tunisia/F-0487/2021	EPI_ISL_8298449
hCoV-19/Tunisia/F-0491/2021	EPI_ISL_8298450
hCoV-19/Tunisia/F-0512/2021	EPI_ISL_8298451
hCoV-19/Tunisia/F-0518/2021	EPI_ISL_8298389
hCoV-19/Tunisia/F-0672/2021	EPI_ISL_8298390
hCoV-19/Tunisia/F-0679/2021	EPI_ISL_8298452
hCoV-19/Tunisia/F-0683/2021	EPI_ISL_8309615
hCoV-19/Tunisia/F-0689/2021	EPI_ISL_8298416
hCoV-19/Tunisia/F-0708/2021	EPI_ISL_8298391
hCoV-19/Tunisia/F-0797/2021	EPI_ISL_8298453
hCoV-19/Tunisia/F-0811/2021	EPI_ISL_8298417
hCoV-19/Tunisia/F-0816/2021	EPI_ISL_8298454
hCoV-19/Tunisia/F-0817/2021	EPI_ISL_8298392
hCoV-19/Tunisia/F-0820/2021	EPI_ISL_8298393
hCoV-19/Tunisia/F-0821/2021	EPI_ISL_8298394
hCoV-19/Tunisia/F-0942/2021	EPI_ISL_8298395
hCoV-19/Tunisia/F-0943/2021	EPI_ISL_8309616
hCoV-19/Tunisia/F-0957/2021	EPI_ISL_8298455

hCoV-19/Tunisia/F-0971/2021	EPI_ISL_8298456
hCoV-19/Tunisia/F-0978/2021	EPI_ISL_8298396
hCoV-19/Tunisia/F-0980/2021	EPI_ISL_8298457
hCoV-19/Tunisia/F-0982/2021	EPI_ISL_8298458
hCoV-19/Tunisia/F-0983/2021	EPI_ISL_8298397
hCoV-19/Tunisia/F-0985/2021	EPI_ISL_8298459
hCoV-19/Tunisia/F-1063/2021	EPI_ISL_8298434
hCoV-19/Tunisia/F-1068/2021	EPI_ISL_8298460
hCoV-19/Tunisia/F-1091/2021	EPI_ISL_8298418
hCoV-19/Tunisia/F-1128/2021	EPI_ISL_8298461
hCoV-19/Tunisia/F-1158/2021	EPI_ISL_8298462
hCoV-19/Tunisia/F-1167/2021	EPI_ISL_8298463
hCoV-19/Tunisia/F-1182/2021	EPI_ISL_8298419
hCoV-19/Tunisia/F-1184/2021	EPI_ISL_8298420
hCoV-19/Tunisia/F-1190/2021	EPI_ISL_8298464
hCoV-19/Tunisia/F-1211/2021	EPI_ISL_8298465
hCoV-19/Tunisia/F-1237/2021	EPI_ISL_8298466
hCoV-19/Tunisia/F-1260/2021	EPI_ISL_8298467
hCoV-19/Tunisia/F-1270/2021	EPI_ISL_8298398
hCoV-19/Tunisia/F-1340/2021	EPI_ISL_8298421
hCoV-19/Tunisia/F-1342/2021	EPI_ISL_8298468
hCoV-19/Tunisia/F-1352/2021	EPI_ISL_8298422
hCoV-19/Tunisia/F-1370/2021	EPI_ISL_8298469
hCoV-19/Tunisia/F-1449/2021	EPI_ISL_8298470
hCoV-19/Tunisia/F-1466/2021	EPI_ISL_8298399
hCoV-19/Tunisia/F-1470/2021	EPI_ISL_8298435
hCoV-19/Tunisia/F-1480/2021	EPI_ISL_8298471
hCoV-19/Tunisia/F-1482/2021	EPI_ISL_8298472
hCoV-19/Tunisia/F-1640/2021	EPI_ISL_8298423
hCoV-19/Tunisia/F-1686/2021	EPI_ISL_8298400
hCoV-19/Tunisia/F-1689/2021	EPI_ISL_8298401
hCoV-19/Tunisia/F-1691/2021	EPI_ISL_8298402
hCoV-19/Tunisia/F-1694/2021	EPI_ISL_8298403
hCoV-19/Tunisia/F-1695/2021	EPI_ISL_8298473
hCoV-19/Tunisia/F-1697/2021	EPI_ISL_8298474
hCoV-19/Tunisia/F-1698/2021	EPI_ISL_8298404
hCoV-19/Tunisia/F-1828/2021	EPI_ISL_8298475
hCoV-19/Tunisia/F-1836/2021	EPI_ISL_8298476
hCoV-19/Tunisia/F-1851/2021	EPI_ISL_8298477
hCoV-19/Tunisia/F-1853/2021	EPI_ISL_8298478
hCoV-19/Tunisia/F-1856/2021	EPI_ISL_8298479
hCoV-19/Tunisia/F-1871/2021	EPI_ISL_8298480
hCoV-19/Tunisia/F-1874/2021	EPI_ISL_8298481
hCoV-19/Tunisia/F-1878/2021	EPI_ISL_8298482
hCoV-19/Tunisia/F-1902/2021	EPI_ISL_8298405
hCoV-19/Tunisia/F-1904/2021	EPI_ISL_8298483
hCoV-19/Tunisia/F-1912/2021	EPI_ISL_8298484
hCoV-19/Tunisia/F-1949/2021	EPI_ISL_8298485

hCoV-19/Tunisia/F-1952/2021	EPI_ISL_8298406
hCoV-19/Tunisia/F-1976/2021	EPI_ISL_8298486
hCoV-19/Tunisia/F-1978/2021	EPI_ISL_8298487
hCoV-19/Tunisia/F-1980/2021	EPI_ISL_8298488
hCoV-19/Tunisia/F-1989/2021	EPI_ISL_8298489
hCoV-19/Tunisia/F-2007/2021	EPI_ISL_8298490
hCoV-19/Tunisia/F-2013/2021	EPI_ISL_8298491
hCoV-19/Tunisia/F-2101/2021	EPI_ISL_8298407
hCoV-19/Tunisia/F-2106/2021	EPI_ISL_8298424
hCoV-19/Tunisia/F-2108/2021	EPI_ISL_8298492
hCoV-19/Tunisia/F-2176/2021	EPI_ISL_8298425
hCoV-19/Tunisia/F-2334/2021	EPI_ISL_8298493
hCoV-19/Tunisia/F-2382/2021	EPI_ISL_8298426
hCoV-19/Tunisia/F-2464/2021	EPI_ISL_8298408
hCoV-19/Tunisia/F-2470/2021	EPI_ISL_8298409
hCoV-19/Tunisia/F-2575/2021	EPI_ISL_8298494
hCoV-19/Tunisia/F-2581/2021	EPI_ISL_8298495
hCoV-19/Tunisia/F-2587/2021	EPI_ISL_8298496
hCoV-19/Tunisia/F-2602/2021	EPI_ISL_8298497
hCoV-19/Tunisia/F-2616/2021	EPI_ISL_8298498
hCoV-19/Tunisia/F-2682/2021	EPI_ISL_8298410
hCoV-19/Tunisia/F-2703/2021	EPI_ISL_8298499
hCoV-19/Tunisia/F-2715/2021	EPI_ISL_8298500
hCoV-19/Tunisia/F-2716/2021	EPI_ISL_8298501
hCoV-19/Tunisia/F-2729/2021	EPI_ISL_8309617
hCoV-19/Tunisia/F-2763/2021	EPI_ISL_8298502
hCoV-19/Tunisia/F-2831/2021	EPI_ISL_8298427
hCoV-19/Tunisia/F-2857/2021	EPI_ISL_8298503
hCoV-19/Tunisia/F-2878/2021	EPI_ISL_8298504
hCoV-19/Tunisia/F-2884/2021	EPI_ISL_8298505
hCoV-19/Tunisia/F-2892/2021	EPI_ISL_8298506
hCoV-19/Tunisia/F-2894/2021	EPI_ISL_8298507
hCoV-19/Tunisia/F-2899/2021	EPI_ISL_8298508
hCoV-19/Tunisia/F-2908/2021	EPI_ISL_8298428
hCoV-19/Tunisia/F-2916/2021	EPI_ISL_8298411
hCoV-19/Tunisia/F-2962/2021	EPI_ISL_8298509
hCoV-19/Tunisia/F-2969/2021	EPI_ISL_8298510
hCoV-19/Tunisia/F-2976/2021	EPI_ISL_8298511
hCoV-19/Tunisia/F-2978/2021	EPI_ISL_8309618
hCoV-19/Tunisia/F-2994/2021	EPI_ISL_8298512
hCoV-19/Tunisia/F-2997/2021	EPI_ISL_8298412
hCoV-19/Tunisia/F-2998/2021	EPI_ISL_8298513
hCoV-19/Tunisia/F-3036/2021	EPI_ISL_8298514
hCoV-19/Tunisia/F-3050/2021	EPI_ISL_8298515
hCoV-19/Tunisia/F-3053/2021	EPI_ISL_8298516
hCoV-19/Tunisia/F-3057/2021	EPI_ISL_8298517
hCoV-19/Tunisia/F-3081/2021	EPI_ISL_8298518
hCoV-19/Tunisia/F-3082/2021	EPI_ISL_8298519

hCoV-19/Tunisia/F-3102/2021	EPI_ISL_8298520
hCoV-19/Tunisia/F-3138/2021	EPI_ISL_8298521
hCoV-19/Tunisia/F-3160/2021	EPI_ISL_8298522
hCoV-19/Tunisia/F-3162/2021	EPI_ISL_8298523
hCoV-19/Tunisia/F-3173/2021	EPI_ISL_8298524
hCoV-19/Tunisia/F-3175/2021	EPI_ISL_8298525
hCoV-19/Tunisia/F-3214/2021	EPI_ISL_8298526
hCoV-19/Tunisia/F-3243/2021	EPI_ISL_8298527
hCoV-19/Tunisia/F-3263/2021	EPI_ISL_8298413
hCoV-19/Tunisia/F-3266/2021	EPI_ISL_8298528
hCoV-19/Tunisia/F-3277/2021	EPI_ISL_8298529
hCoV-19/Tunisia/F-3334/2021	EPI_ISL_8298530
hCoV-19/Tunisia/F-3344/2021	EPI_ISL_8298531
hCoV-19/Tunisia/F-3350/2021	EPI_ISL_8298532
hCoV-19/Tunisia/F-3399/2021	EPI_ISL_8298533
hCoV-19/Tunisia/F-3411/2021	EPI_ISL_8298534
hCoV-19/Tunisia/F-3422/2021	EPI_ISL_8298535
hCoV-19/Tunisia/F-3452/2021	EPI_ISL_8298536
hCoV-19/Tunisia/F-3464/2021	EPI_ISL_8298537
hCoV-19/Tunisia/F-3469/2021	EPI_ISL_8298538
hCoV-19/Tunisia/F-3486/2021	EPI_ISL_8298539
hCoV-19/Tunisia/F-3530/2021	EPI_ISL_8298414
hCoV-19/Tunisia/F-3539/2021	EPI_ISL_8298540
hCoV-19/Tunisia/F-3571/2021	EPI_ISL_8298415
hCoV-19/Tunisia/F-3576/2021	EPI_ISL_8298432
hCoV-19/Tunisia/F-3577/2021	EPI_ISL_8298541
hCoV-19/Tunisia/F-3627/2021	EPI_ISL_8298542
hCoV-19/Tunisia/F-3651/2021	EPI_ISL_8298543
hCoV-19/Tunisia/F-3693/2021	EPI_ISL_8298544
hCoV-19/Tunisia/F-3710/2021	EPI_ISL_8298545
hCoV-19/Tunisia/F-3723/2021	EPI_ISL_8298546
hCoV-19/Tunisia/F-3746/2021	EPI_ISL_8298547
hCoV-19/Tunisia/F-3760/2021	EPI_ISL_8298548
hCoV-19/Tunisia/F-3837/2021	EPI_ISL_8298549
hCoV-19/Tunisia/F-3965/2021	EPI_ISL_8298550
hCoV-19/Tunisia/F-4000/2021	EPI_ISL_8298551
hCoV-19/Tunisia/F-4003/2021	EPI_ISL_8309619
hCoV-19/Tunisia/F-4081/2021	EPI_ISL_11349037
hCoV-19/Tunisia/F-4279/2021	EPI_ISL_11333852
hCoV-19/Tunisia/F-4392/2021	EPI_ISL_11333853
hCoV-19/Tunisia/F-4397/2021	EPI_ISL_11333854
hCoV-19/Tunisia/F-4532/2021	EPI_ISL_11333855
hCoV-19/Tunisia/F-4572/2021	EPI_ISL_11333856
hCoV-19/Tunisia/F-4584/2021	EPI_ISL_11333857
hCoV-19/Tunisia/F-4610/2021	EPI_ISL_11333858
hCoV-19/Tunisia/F-4697/2021	EPI_ISL_11333859
hCoV-19/Tunisia/F-4722/2021	EPI_ISL_11333860
hCoV-19/Tunisia/F-4762/2021	EPI_ISL_11333861

hCoV-19/Tunisia/F-4820/2021	EPI_ISL_11333862
hCoV-19/Tunisia/F-4878/2021	EPI_ISL_11333863
hCoV-19/Tunisia/F-4879/2021	EPI_ISL_11333864
hCoV-19/Tunisia/F-4881/2021	EPI_ISL_11333865
hCoV-19/Tunisia/F-4882/2021	EPI_ISL_11333866
hCoV-19/Tunisia/S-0502/2021	EPI_ISL_11172692
hCoV-19/Tunisia/S-0519/2021	EPI_ISL_10141433
hCoV-19/Tunisia/S0525/2021	EPI_ISL_11172693
hCoV-19/Tunisia/S0526/2021	EPI_ISL_11172694
hCoV-19/Tunisia/S0527/2021	EPI_ISL_11172695
hCoV-19/Tunisia/S0529/2021	EPI_ISL_11172696
hCoV-19/Tunisia/S-0702/2021	EPI_ISL_10141447
hCoV-19/Tunisia/S-0704/2021	EPI_ISL_10141446
hCoV-19/Tunisia/S-0705/2021	EPI_ISL_10141539
hCoV-19/Tunisia/S-0709/2021	EPI_ISL_10141543
hCoV-19/Tunisia/S-0714/2021	EPI_ISL_10141545
hCoV-19/Tunisia/S-0716/2021	EPI_ISL_10141443
hCoV-19/Tunisia/S-0718/2021	EPI_ISL_10141395
hCoV-19/Tunisia/S-0721/2021	EPI_ISL_10141400
hCoV-19/Tunisia/S-0722/2021	EPI_ISL_10141393
hCoV-19/Tunisia/S-0724/2021	EPI_ISL_10141392
hCoV-19/Tunisia/S-0738/2021	EPI_ISL_10141410
hCoV-19/Tunisia/S-0739/2021	EPI_ISL_10141398
hCoV-19/Tunisia/S-0746/2021	EPI_ISL_10141390
hCoV-19/Tunisia/S-0750/2021	EPI_ISL_10141397
hCoV-19/Tunisia/S-0759/2021	EPI_ISL_10141516
hCoV-19/Tunisia/S-0762/2021	EPI_ISL_10141442
hCoV-19/Tunisia/S-0802/2021	EPI_ISL_10141530
hCoV-19/Tunisia/S-0829/2021	EPI_ISL_10141396
hCoV-19/Tunisia/S-0847/2021	EPI_ISL_10141459
hCoV-19/Tunisia/S-0893/2021	EPI_ISL_10141402
hCoV-19/Tunisia/S-1035/2021	EPI_ISL_8298436
hCoV-19/Tunisia/S-1036/2021	EPI_ISL_8298437
hCoV-19/Tunisia/S-1037/2021	EPI_ISL_8298438
hCoV-19/Tunisia/S-1038/2021	EPI_ISL_8298441
hCoV-19/Tunisia/S-1039/2021	EPI_ISL_8309614
hCoV-19/Tunisia/S-1040/2021	EPI_ISL_8298442
hCoV-19/Tunisia/S-1041/2021	EPI_ISL_8298443
hCoV-19/Tunisia/S-1045/2021	EPI_ISL_11333867
hCoV-19/Tunisia/S-1047/2021	EPI_ISL_11333869
hCoV-19/Tunisia/S-1050/2021	EPI_ISL_11349039
hCoV-19/Tunisia/S-1052/2021	EPI_ISL_11333870
hCoV-19/Tunisia/S-1053/2021	EPI_ISL_11333871
hCoV-19/Tunisia/S-1054/2021	EPI_ISL_11333872
hCoV-19/Tunisia/S-1055/2021	EPI_ISL_11333873
hCoV-19/Tunisia/S-1056/2021	EPI_ISL_11333874
hCoV-19/Tunisia/S-1059/2021	EPI_ISL_11333876
hCoV-19/Tunisia/S-1060/2021	EPI_ISL_11333877

hCoV-19/Tunisia/S-1061/2021	EPI_ISL_11333878
hCoV-19/Tunisia/S-1065/2021	EPI_ISL_11333881
hCoV-19/Tunisia/S-1067/2021	EPI_ISL_11333882
hCoV-19/Tunisia/S-1068/2021	EPI_ISL_11333883
hCoV-19/Tunisia/S-1069/2021	EPI_ISL_11333884
hCoV-19/Tunisia/S-1070/2021	EPI_ISL_11333885
hCoV-19/Tunisia/S-1071/2021	EPI_ISL_11333886
hCoV-19/Tunisia/S-1072/2021	EPI_ISL_11333887
hCoV-19/Tunisia/S-1073/2021	EPI_ISL_11333888
hCoV-19/Tunisia/S-1074/2021	EPI_ISL_11333889
hCoV-19/Tunisia/S-1076/2021	EPI_ISL_11333891
hCoV-19/Tunisia/S-1077/2021	EPI_ISL_11333892
hCoV-19/Tunisia/S-1078/2021	EPI_ISL_11333893
hCoV-19/Tunisia/S-1080/2021	EPI_ISL_11333894
hCoV-19/Tunisia/S-1082/2021	EPI_ISL_11333896
hCoV-19/Tunisia/S-1083/2021	EPI_ISL_11333897
hCoV-19/Tunisia/S-1085/2021	EPI_ISL_11333898
hCoV-19/Tunisia/S-1088/2021	EPI_ISL_11333900
hCoV-19/Tunisia/S-1094/2021	EPI_ISL_11333901
hCoV-19/Tunisia/S-1100/2021	EPI_ISL_11333903
hCoV-19/Tunisia/S-1103/2021	EPI_ISL_11349040
hCoV-19/Tunisia/S-1105/2021	EPI_ISL_11333904
hCoV-19/Tunisia/S-1107/2021	EPI_ISL_11333905
hCoV-19/Tunisia/S-1108/2021	EPI_ISL_11333906
hCoV-19/Tunisia/S-1110/2021	EPI_ISL_11333907
hCoV-19/Tunisia/S-1111/2021	EPI_ISL_11333908
hCoV-19/Tunisia/S-1112/2021	EPI_ISL_11333909
hCoV-19/Tunisia/S-1115/2021	EPI_ISL_11333910
hCoV-19/Tunisia/S-1118/2021	EPI_ISL_11333911
hCoV-19/Tunisia/S-1121/2021	EPI_ISL_11333912
hCoV-19/Tunisia/S-1123/2021	EPI_ISL_11333913
hCoV-19/Tunisia/S-1124/2021	EPI_ISL_11333914
hCoV-19/Tunisia/S-1126/2021	EPI_ISL_11333915
hCoV-19/Tunisia/S-1129/2021	EPI_ISL_11333916
hCoV-19/Tunisia/S-1130/2021	EPI_ISL_11333917
hCoV-19/Tunisia/S-1132/2021	EPI_ISL_11333918
hCoV-19/Tunisia/S-1137/2021	EPI_ISL_11333919
hCoV-19/Tunisia/S-1138/2021	EPI_ISL_11333920
hCoV-19/Tunisia/S-1142/2021	EPI_ISL_11333921
hCoV-19/Tunisia/S-1152/2021	EPI_ISL_11333922
hCoV-19/Tunisia/S-1153/2021	EPI_ISL_11333923
hCoV-19/Tunisia/S-1154/2021	EPI_ISL_11333924
hCoV-19/Tunisia/S-1167/2021	EPI_ISL_11349041
hCoV-19/Tunisia/S-1176/2021	EPI_ISL_11333926
hCoV-19/Tunisia/S-1188/2021	EPI_ISL_11333928
hCoV-19/Tunisia/S-1192/2021	EPI_ISL_11333929
hCoV-19/Tunisia/S-1194/2021	EPI_ISL_11333930
hCoV-19/Tunisia/S-1196/2021	EPI_ISL_11333931

hCoV-19/Tunisia/S-1200/2021	EPI_ISL_11333932
hCoV-19/Tunisia/S-1204/2021	EPI_ISL_11333933
hCoV-19/Tunisia/S-1205/2021	EPI_ISL_11333934
hCoV-19/Tunisia/S-1206/2021	EPI_ISL_11333935
hCoV-19/Tunisia/S-1208/2021	EPI_ISL_11333936
hCoV-19/Tunisia/S-1213/2021	EPI_ISL_11333937
hCoV-19/Tunisia/S-1217/2021	EPI_ISL_11333938
hCoV-19/Tunisia/S-1224/2021	EPI_ISL_11333939
hCoV-19/Tunisia/S-1234/2021	EPI_ISL_11333940
hCoV-19/Tunisia/S-1235/2021	EPI_ISL_11333941
hCoV-19/Tunisia/S-1237/2021	EPI_ISL_11333942
hCoV-19/Tunisia/S-1244/2021	EPI_ISL_11333944
hCoV-19/Tunisia/S-1275/2021	EPI_ISL_11333946
hCoV-19/Tunisia/S-1276/2021	EPI_ISL_11333947
hCoV-19/Tunisia/S-1277/2021	EPI_ISL_11333948
hCoV-19/Tunisia/S-1283/2021	EPI_ISL_11333949
hCoV-19/Tunisia/S-1285/2021	EPI_ISL_11333950
hCoV-19/Tunisia/S-1287/2021	EPI_ISL_11333952
hCoV-19/Tunisia/S-1294/2021	EPI_ISL_11333953
hCoV-19/Tunisia/S-1298/2021	EPI_ISL_11333954
hCoV-19/Tunisia/S-1299/2021	EPI_ISL_11333955
hCoV-19/Tunisia/S-1300/2021	EPI_ISL_11333956
hCoV-19/Tunisia/S-1303/2021	EPI_ISL_11333957
hCoV-19/Tunisia/S-1304/2021	EPI_ISL_11333958
hCoV-19/Tunisia/S-1307/2021	EPI_ISL_11349042
hCoV-19/Tunisia/S-1308/2021	EPI_ISL_11333959
hCoV-19/Tunisia/S-1309/2021	EPI_ISL_11333960
hCoV-19/Tunisia/S-1311/2021	EPI_ISL_11333961
hCoV-19/Tunisia/S-1312/2021	EPI_ISL_11333962
hCoV-19/Tunisia/S-1313/2021	EPI_ISL_11333963
hCoV-19/Tunisia/S-1314/2021	EPI_ISL_11333964
hCoV-19/Tunisia/S-1315/2021	EPI_ISL_11333965
hCoV-19/Tunisia/S-1319/2021	EPI_ISL_11333966
hCoV-19/Tunisia/S-1321/2021	EPI_ISL_11333967
hCoV-19/Tunisia/S-1325/2021	EPI_ISL_11333968
hCoV-19/Tunisia/S-1327/2021	EPI_ISL_11333969
hCoV-19/Tunisia/S-1332/2021	EPI_ISL_11333970
hCoV-19/Tunisia/S-1333/2021	EPI_ISL_11333971
hCoV-19/Tunisia/S-1336/2021	EPI_ISL_11333972
hCoV-19/Tunisia/S-1338/2021	EPI_ISL_11333973
hCoV-19/Tunisia/S-1339/2021	EPI_ISL_11333974
hCoV-19/Tunisia/S-1342/2021	EPI_ISL_11333975
hCoV-19/Tunisia/S-1343/2021	EPI_ISL_11349043
hCoV-19/Tunisia/S-1345/2021	EPI_ISL_11333976
hCoV-19/Tunisia/S-1346/2021	EPI_ISL_11333977
hCoV-19/Tunisia/S-1347/2021	EPI_ISL_11333978
hCoV-19/Tunisia/S-1349/2021	EPI_ISL_11333979
hCoV-19/Tunisia/S-1350/2021	EPI_ISL_11333980

hCoV-19/Tunisia/S-1351/2021	EPI_ISL_11333981
hCoV-19/Tunisia/S-1353/2021	EPI_ISL_11333982
hCoV-19/Tunisia/S-1354/2021	EPI_ISL_11333983
hCoV-19/Tunisia/S-1356/2021	EPI_ISL_11333984
hCoV-19/Tunisia/S-1357/2021	EPI_ISL_11333985
hCoV-19/Tunisia/S-1358/2021	EPI_ISL_11333986
hCoV-19/Tunisia/S-1359/2021	EPI_ISL_11333987
hCoV-19/Tunisia/S-1360/2021	EPI_ISL_11333988
hCoV-19/Tunisia/S-1361/2021	EPI_ISL_11333989
hCoV-19/Tunisia/S-1362/2021	EPI_ISL_11333990
hCoV-19/Tunisia/S-1363/2021	EPI_ISL_11333991
hCoV-19/Tunisia/S-1364/2021	EPI_ISL_11333992
hCoV-19/Tunisia/S-1366/2021	EPI_ISL_11349044
hCoV-19/Tunisia/S-1370/2021	EPI_ISL_11333994
hCoV-19/Tunisia/S-1371/2021	EPI_ISL_11333995
hCoV-19/Tunisia/S-1378/2021	EPI_ISL_11333997
hCoV-19/Tunisia/S-1381/2021	EPI_ISL_11333999
hCoV-19/Tunisia/S-1382/2021	EPI_ISL_11334000
hCoV-19/Tunisia/S-1385/2021	EPI_ISL_11334002
hCoV-19/Tunisia/S-1390/2021	EPI_ISL_11334004
hCoV-19/Tunisia/S-1399/2021	EPI_ISL_11334006
hCoV-19/Tunisia/S-1408/2021	EPI_ISL_11334008
hCoV-19/Tunisia/S-1423/2021	EPI_ISL_11334014
hCoV-19/Tunisia/S-1425/2021	EPI_ISL_11334016
hCoV-19/Tunisia/S-1426/2021	EPI_ISL_11334017
hCoV-19/Tunisia/S-1428/2021	EPI_ISL_11334018
hCoV-19/Tunisia/S-1431/2021	EPI_ISL_11334019
hCoV-19/Tunisia/S-1432/2021	EPI_ISL_11334020
hCoV-19/Tunisia/S-1433/2021	EPI_ISL_11334021
hCoV-19/Tunisia/S-1434/2021	EPI_ISL_11334022
hCoV-19/Tunisia/S-1437/2021	EPI_ISL_11349046
hCoV-19/Tunisia/S-1439/2021	EPI_ISL_11334025
hCoV-19/Tunisia/S-1442/2021	EPI_ISL_11334026
hCoV-19/Tunisia/S-1449/2021	EPI_ISL_11334030
hCoV-19/Tunisia/S-1453/2021	EPI_ISL_11349047
hCoV-19/Tunisia/S-1454/2021	EPI_ISL_11334031
hCoV-19/Tunisia/S-1460/2021	EPI_ISL_11334033
hCoV-19/Tunisia/S-1462/2021	EPI_ISL_11334034
hCoV-19/Tunisia/S-1463/2021	EPI_ISL_11334035
hCoV-19/Tunisia/S-1469/2021	EPI_ISL_11334037
hCoV-19/Tunisia/S-1470/2021	EPI_ISL_11349048
hCoV-19/Tunisia/S-1472/2021	EPI_ISL_11334038
hCoV-19/Tunisia/S-1473/2021	EPI_ISL_11334039
hCoV-19/Tunisia/S-1474/2021	EPI_ISL_11334040
hCoV-19/Tunisia/S-1477/2021	EPI_ISL_11334041
hCoV-19/Tunisia/S-1478/2021	EPI_ISL_11334042
hCoV-19/Tunisia/S-1479/2021	EPI_ISL_11334043
hCoV-19/Tunisia/S-1480/2021	EPI_ISL_11334044

hCoV-19/Tunisia/S-1482/2021	EPI_ISL_11334045
hCoV-19/Tunisia/S-1484/2021	EPI_ISL_11334046
hCoV-19/Tunisia/S-1486/2021	EPI_ISL_11334047
hCoV-19/Tunisia/S-1505/2021	EPI_ISL_11334049
hCoV-19/Tunisia/S-1508/2021	EPI_ISL_11349049
hCoV-19/Tunisia/S-1509/2021	EPI_ISL_11349050
hCoV-19/Tunisia/S-1510/2021	EPI_ISL_11349051
hCoV-19/Tunisia/S-1511/2021	EPI_ISL_11349052
hCoV-19/Tunisia/S-1512/2021	EPI_ISL_11334050
hCoV-19/Tunisia/S-1513/2021	EPI_ISL_11349053
hCoV-19/Tunisia/S-1516/2021	EPI_ISL_11334051
hCoV-19/Tunisia/S-1519/2021	EPI_ISL_11334052
hCoV-19/Tunisia/S-1524/2021	EPI_ISL_11334053
hCoV-19/Tunisia/S-1529/2021	EPI_ISL_11349055
hCoV-19/Tunisia/S-1531/2021	EPI_ISL_11334057
hCoV-19/Tunisia/S-1532/2021	EPI_ISL_11349056
hCoV-19/Tunisia/S-1534/2021	EPI_ISL_11349057
hCoV-19/Tunisia/S-1535/2021	EPI_ISL_11349058
hCoV-19/Tunisia/S-1538/2021	EPI_ISL_11349059
hCoV-19/Tunisia/S-1539/2021	EPI_ISL_11334061
hCoV-19/Tunisia/S-1543/2021	EPI_ISL_11349060
hCoV-19/Tunisia/S-1544/2021	EPI_ISL_11349061
hCoV-19/Tunisia/S-1545/2021	EPI_ISL_11349062
hCoV-19/Tunisia/S-1547/2021	EPI_ISL_11334063
hCoV-19/Tunisia/S-1548/2021	EPI_ISL_11349064
hCoV-19/Tunisia/S-1549/2021	EPI_ISL_11334064
hCoV-19/Tunisia/S-1550/2021	EPI_ISL_11349065
hCoV-19/Tunisia/S-1554/2021	EPI_ISL_11334067
hCoV-19/Tunisia/S-1555/2021	EPI_ISL_11334068
hCoV-19/Tunisia/S-1556/2021	EPI_ISL_11334069
hCoV-19/Tunisia/S-1557/2021	EPI_ISL_11334070
hCoV-19/Tunisia/S-1558/2021	EPI_ISL_11334071
hCoV-19/Tunisia/S-1559/2021	EPI_ISL_11334072
hCoV-19/Tunisia/S-1560/2021	EPI_ISL_11334073
hCoV-19/Tunisia/S-1561/2021	EPI_ISL_11349066
hCoV-19/Tunisia/S-1562/2021	EPI_ISL_11334074
hCoV-19/Tunisia/S-1563/2021	EPI_ISL_11334075
hCoV-19/Tunisia/S-1564/2021	EPI_ISL_11334076
hCoV-19/Tunisia/S-1565/2021	EPI_ISL_11334077
hCoV-19/Tunisia/S-1566/2021	EPI_ISL_11334078
hCoV-19/Tunisia/S-1567/2021	EPI_ISL_11334079
hCoV-19/Tunisia/S-1568/2021	EPI_ISL_11334080
hCoV-19/Tunisia/S-1576/2021	EPI_ISL_11334081
hCoV-19/Tunisia/U-9630/2021	EPI_ISL_10141551
hCoV-19/Tunisia/V-0679/2021	EPI_ISL_8298552
hCoV-19/Tunisia/V-0793/2021	EPI_ISL_8298429
hCoV-19/Tunisia/V-1052/2021	EPI_ISL_8298433
hCoV-19/Tunisia/V-1102/2021	EPI_ISL_8298440

hCoV-19/Tunisia/V-1165/2021	EPI_ISL_8298553
hCoV-19/Tunisia/V-1490/2021	EPI_ISL_8298554
hCoV-19/Tunisia/V-1853/2021	EPI_ISL_8298430
hCoV-19/Tunisia/V-1932/2021	EPI_ISL_8298431
hCoV-19/Tunisia/V-2116/2021	EPI_ISL_8298439
hCoV-19/Tunisia/V-2179/2021	EPI_ISL_8309620
hCoV-19/Tunisia/V-2405/2021	EPI_ISL_11334083
hCoV-19/Tunisia/S-0568/2021	EPI_ISL_11881880
hCoV-19/Tunisia/S-0570/2021	EPI_ISL_11881881
hCoV-19/Tunisia/S-0571/2021	EPI_ISL_11881882
hCoV-19/Tunisia/S-0572/2021	EPI_ISL_11881883
hCoV-19/Tunisia/S-0573/2021	EPI_ISL_11881884
hCoV-19/Tunisia/S-0575/2021	EPI_ISL_11881885
hCoV-19/Tunisia/S-0577/2021	EPI_ISL_11881886
hCoV-19/Tunisia/S-0579/2021	EPI_ISL_11881887
hCoV-19/Tunisia/S-0580/2021	EPI_ISL_11881888
hCoV-19/Tunisia/S-0581/2021	EPI_ISL_11881889
hCoV-19/Tunisia/S-0582/2021	EPI_ISL_11881890
hCoV-19/Tunisia/S-0584/2021	EPI_ISL_11881891
hCoV-19/Tunisia/S-0585/2021	EPI_ISL_11881892
hCoV-19/Tunisia/S-0586/2021	EPI_ISL_11881893
hCoV-19/Tunisia/S-0587/2021	EPI_ISL_11881894
hCoV-19/Tunisia/S-0589/2021	EPI_ISL_11881895
hCoV-19/Tunisia/S-0591/2021	EPI_ISL_11881896
hCoV-19/Tunisia/S-0594/2021	EPI_ISL_11881897
hCoV-19/Tunisia/S-0596/2021	EPI_ISL_11881898
hCoV-19/Tunisia/S-0597/2021	EPI_ISL_11881899
hCoV-19/Tunisia/S-0598/2021	EPI_ISL_11881900
hCoV-19/Tunisia/S-0599/2021	EPI_ISL_11881901
hCoV-19/Tunisia/S-0600/2021	EPI_ISL_11881902
hCoV-19/Tunisia/S-0602/2021	EPI_ISL_11881903
hCoV-19/Tunisia/S-0645/2021	EPI_ISL_11881904
hCoV-19/Tunisia/S-0674/2021	EPI_ISL_11881905
hCoV-19/Tunisia/S-0676/2021	EPI_ISL_11881906
hCoV-19/Tunisia/S-0715/2021	EPI_ISL_11881907
hCoV-19/Tunisia/S-0733/2021	EPI_ISL_11881908
hCoV-19/Tunisia/S-0740/2021	EPI_ISL_11881909
hCoV-19/Tunisia/S-0748/2021	EPI_ISL_11881910
hCoV-19/Tunisia/S-0797/2021	EPI_ISL_11881911
hCoV-19/Tunisia/S-0799/2021	EPI_ISL_11881912
hCoV-19/Tunisia/S-0800/2021	EPI_ISL_11881913
hCoV-19/Tunisia/S-0806/2021	EPI_ISL_11881914
hCoV-19/Tunisia/S-0812/2021	EPI_ISL_11881915
hCoV-19/Tunisia/S-0816/2021	EPI_ISL_11881916
hCoV-19/Tunisia/S-0818/2021	EPI_ISL_11881917
hCoV-19/Tunisia/S-0819/2021	EPI_ISL_11881918
hCoV-19/Tunisia/S-0820/2021	EPI_ISL_11881919
hCoV-19/Tunisia/S-0821/2021	EPI_ISL_11881920
hCoV-19/Tunisia/S-0826/2021	EPI_ISL_11881921
hCoV-19/Tunisia/S-0853/2021	EPI_ISL_11881922

hCoV-19/Tunisia/S-0862/2021	EPI_ISL_11881923
hCoV-19/Tunisia/S-0871/2021	EPI_ISL_11881924
hCoV-19/Tunisia/S-0875/2021	EPI_ISL_11881925
hCoV-19/Tunisia/S-0879/2021	EPI_ISL_11881926
hCoV-19/Tunisia/S-0899/2021	EPI_ISL_11881927
hCoV-19/Tunisia/S-0901/2021	EPI_ISL_11881928
