# Supplementary material for: The Delta variant wave in Tunisia: Genetic diversity, spatio-temporal distribution and evidence of the spread of a divergent AY.122 sub-lineage
Source: Front Public Health. 2023 Jan 4;10:990832. doi: 10.3389/fpubh.2022.990832 (PMC9846204; doi:10.3389/fpubh.2022.990832)
Supplement: Supplementary file 2 [file Data_Sheet_2.PDF]

The Delta variant wave in Tunisia: Genetic diversity, spatio-temporal distribution and evidence of the spread of a divergent AY.122 sub-lineage

Sondes Haddad-Boubaker[1,2,3*], Marwa Arbi†[1,4], Oussema Souiai†[4], Anissa Chouikha[1,2,3], Wasfi Fares[1,2,3], Maha Mastouri[5], Hela Karray[6], Olfa Bahri[7], Halim Trabelsi[8], Naila Hannachi[9], Yassine Chaabouni[10],Hanène Smaoui[11, 12], Sophia Besbes Bouhalila[13], Soumaya Foughali[14], Mariem Zribi[15], Mariem Gdoura[1,2,3,16], Asma Lamari[1,2], Henda Touzi[1,2,3], Mouna Safer[17], Nissaf Ben Alaya[17], Alia Ben Kahla[4],IlhemBoutiba Ben Boubaker[18, 19], Henda Triki[1,2,3].

Supplementary Table2. Bayes Factors for the identified transitions of AY.122 variant between different Tunisian governorates

Transition	BF
Tunis - Ariana	1376534.83
Tunis - Manouba	12.04
Nabeul - Gafsa	11.84
Tunis - Nabeul	12132.57
Nabeul - Manouba	5.75
Nabeul - Kairouan	3.01
Nabeul - Tataouin	2.58
Kairouan - Zaghouane	39.96
Ben Arous - Tunis	1376534.83
Tunis - Monastir	7.43
Manouba - Jendouba	86.23
Kairouan - Sfax	78.39
Kairouan - Siliana	201.58
Kairouan - Jendouba	117.36
Kairouan - Sousse	43.74
Tunis - Sousse	5.24
Tunis - Kef	28.31
Nabeul - Medenine	259.69
