# Supplementary material for: The Delta variant wave in Tunisia: Genetic diversity, spatio-temporal distribution and evidence of the spread of a divergent AY.122 sub-lineage
Source: Front Public Health. 2023 Jan 4;10:990832. doi: 10.3389/fpubh.2022.990832 (PMC9846204; doi:10.3389/fpubh.2022.990832)
Supplement: Supplementary file 4 [file Data_Sheet_4.PDF]

Article Title: The Delta variant wave in Tunisia: Genetic diversity, spatio-temporal distribution and evidence of the spread of a divergent AY.122 sub-lineage

Authors: Sondes Haddad-Boubaker[1,2,3*], Marwa Arbi†[1,4], Oussema Souiai†[4], Anissa Chouikha[1,2,3], Wasfi Fares[1,2,3], Maha Mastouri[5], Hela Karray[6], Olfa Bahri[7], Halim Trabelsi[8], Naila Hannachi[9], Yassine Chaabouni[10],Hanène Smaoui[11, 12], Sophia Besbes Bouhalila[13], Soumaya Foughali[14], Mariem Zribi[15], Mariem Gdoura[1,2,3,16], Asma Lamari[1,2], Henda Touzi[1,2,3], Mouna Safer[17], Nissaf Ben Alaya[17], Alia Ben Kahla[4],IlhemBoutiba Ben Boubaker[18, 19], Henda Triki[1,2,3].

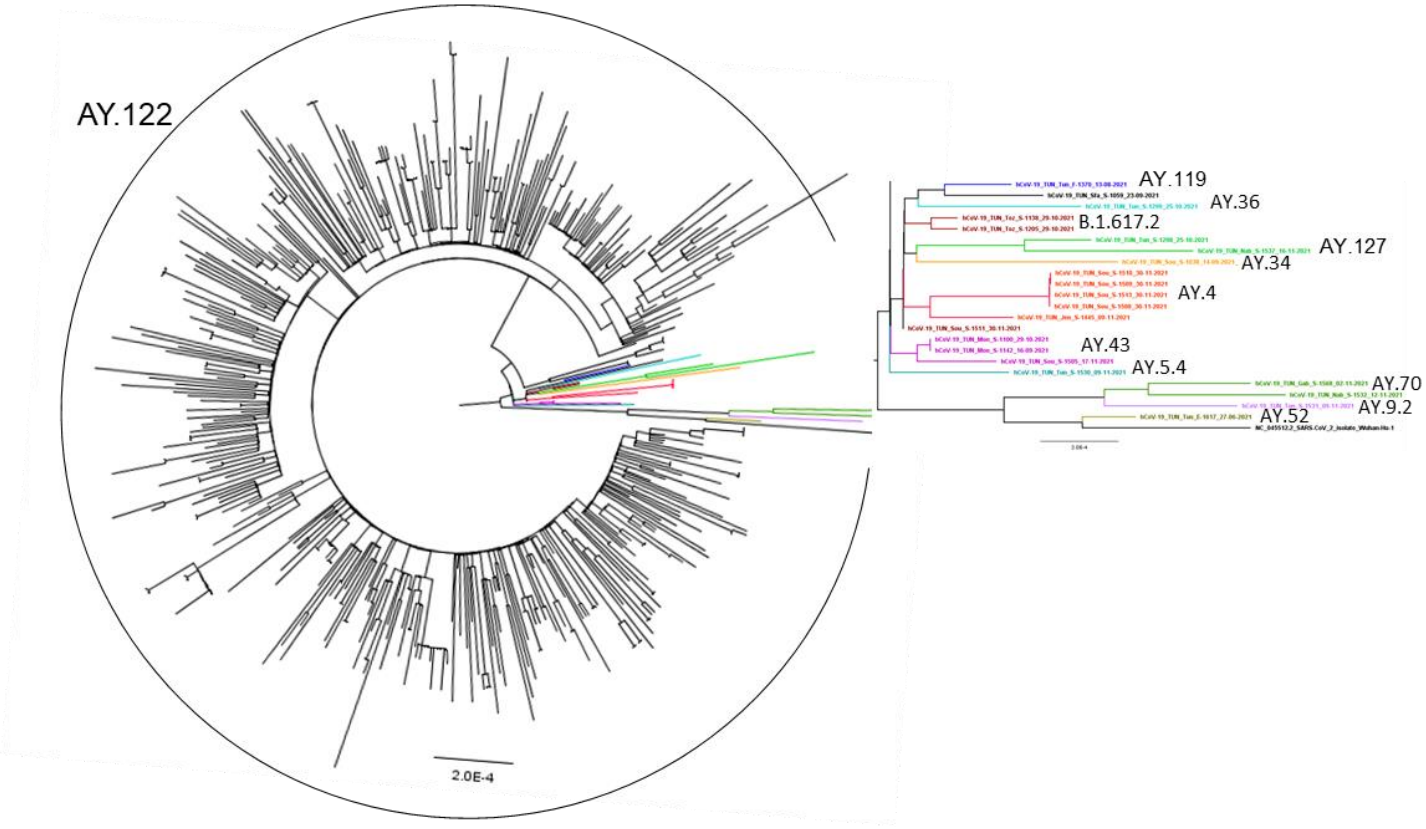

Supplementary Figure2. Phylogenetic tree of the 468 SARS-CoV-2 genomes from Tunisia, May-December 2021. The Maximum likelihood tree, with a bootstrap replication of 1000 cycles, shows distribution of Tunisian strains among 12 sub-lineages of Delta variant (AY.122, B.1.617.2, AY.4, AY.43, AY.70, AY.127, AY.9.2, AY.52, AY.5.4, AY.36, AY.34, AY.119) and predominance of AY.122 sub-lineage. The representative pangolin lineages are marked in different colors. FigTree v1.4.4 were used to visualize and edit the generated tree.
