# Supplementary material for: The Delta variant wave in Tunisia: Genetic diversity, spatio-temporal distribution and evidence of the spread of a divergent AY.122 sub-lineage
Source: Front Public Health. 2023 Jan 4;10:990832. doi: 10.3389/fpubh.2022.990832 (PMC9846204; doi:10.3389/fpubh.2022.990832)
Supplement: Supplementary file 5 [file Data_Sheet_5.PDF]

The Delta variant wave in Tunisia: Genetic diversity, spatio-temporal distribution and evidence of the spread of a divergent AY.122 sub-lineage

Sondes Haddad-Boubaker[1,2,3*], Marwa Arbi†[1,4], Oussema Souiai†[4], Anissa Chouikha[1,2,3], Wasfi Fares[1,2,3], Maha Mastouri[5], Hela Karray[6], Olfa Bahri[7], Halim Trabelsi[8], Naila Hannachi[9], Yassine Chaabouni[10],Hanène Smaoui[11, 12], Sophia Besbes Bouhalila[13], Soumaya Foughali[14], Mariem Zribi[15], Mariem Gdoura[1,2,3,16], Asma Lamari[1,2], Henda Touzi[1,2,3], Mouna Safer[17], Nissaf Ben Alaya[17], Alia Ben Kahla[4],IlhemBoutiba Ben Boubaker[18, 19], Henda Triki[1,2,3].

Supplementary Table3. Bayes Factors for the identified transitions of AY.122 variant between different Tunisian regions

**Transition**	**BF**
Tunis region - North	294254.38
Tunis region - Sahel	104.44
North - Center	77.06
North - South	9.38
Center - Sahel	269.06
