# Supplementary material for: The Delta variant wave in Tunisia: Genetic diversity, spatio-temporal distribution and evidence of the spread of a divergent AY.122 sub-lineage
Source: Front Public Health. 2023 Jan 4;10:990832. doi: 10.3389/fpubh.2022.990832 (PMC9846204; doi:10.3389/fpubh.2022.990832)
Supplement: Supplementary file 6 [file Data_Sheet_6.PDF]

**Article Title: The Delta variant wave in Tunisia: Genetic diversity, spatio-temporal distribution and evidence of the spread of a divergent AY.122 sub-lineage**

**Authors**: Sondes Haddad-Boubaker[1,2,3*], Marwa Arbi†[1,4], Oussema Souiai†[4], Anissa Chouikha[1,2,3], Wasfi Fares[1,2,3], Maha Mastouri[5], Hela Karray[6], Olfa Bahri[7], Halim Trabelsi[8], Naila Hannachi[9], Yassine Chaabouni[10],Hanène Smaoui[11, 12], Sophia Besbes Bouhalila[13], Soumaya Foughali[14], Mariem Zribi[15], Mariem Gdoura[1,2,3,16], Asma Lamari[1,2], Henda Touzi[1,2,3], Mouna Safer[17], Nissaf Ben Alaya[17], Alia Ben Kahla[4],IlhemBoutiba Ben Boubaker[18, 19], Henda Triki[1,2,3].

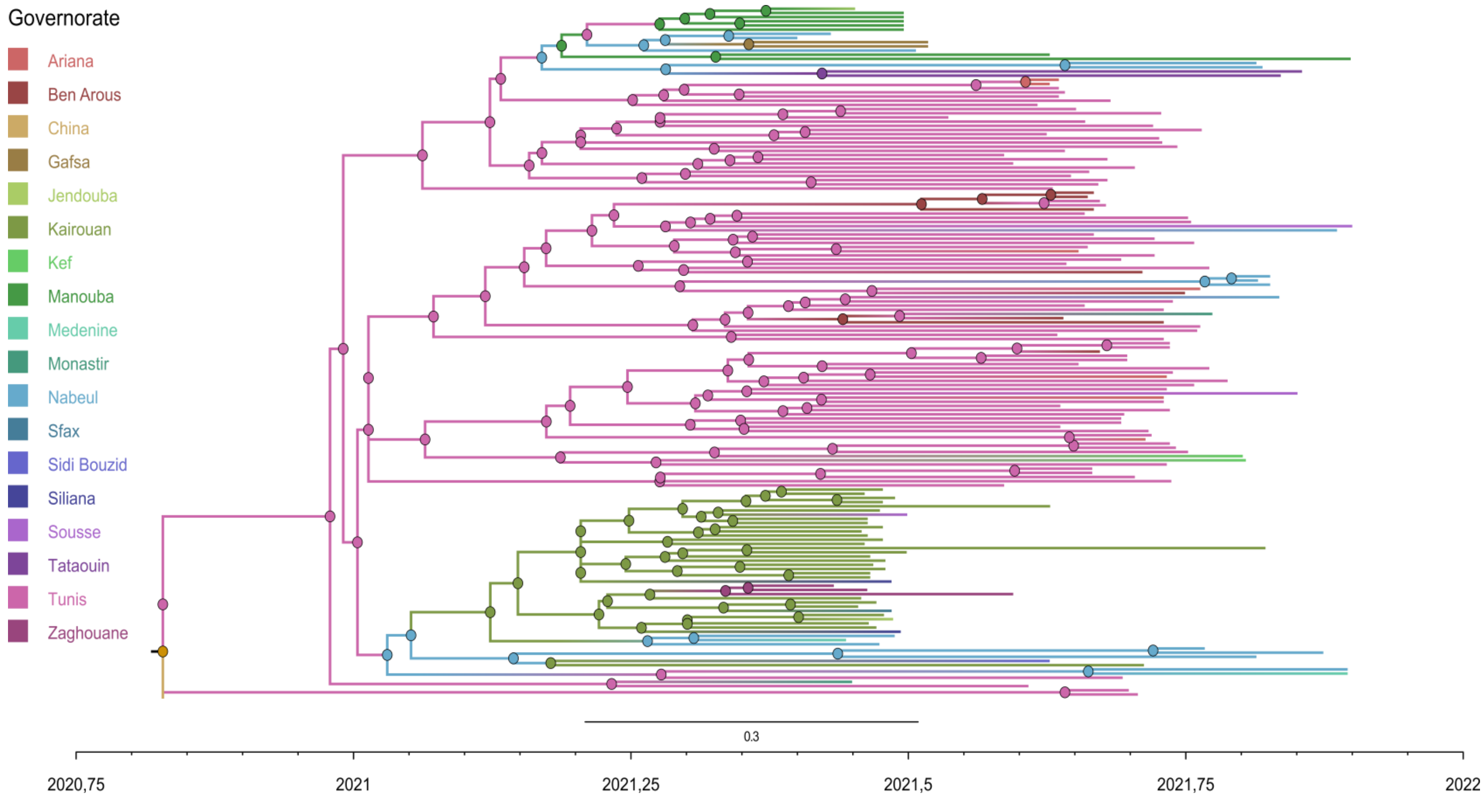


**Supplementary Figure4**. Time-scaled Maximum Clade Credibility tree of AY.122 Sub-lineage in Tunisia. Strains from different governorates are marked in different colors as indicated in the legend. Tree were visualized using Figtree software.
