# Supplementary figures and images for: The Delta variant wave in Tunisia: Genetic diversity, spatio-temporal distribution and evidence of the spread of a divergent AY.122 sub-lineage
Source: Front Public Health. 2023 Jan 4;10:990832. doi: 10.3389/fpubh.2022.990832 (PMC9846204; doi:10.3389/fpubh.2022.990832)

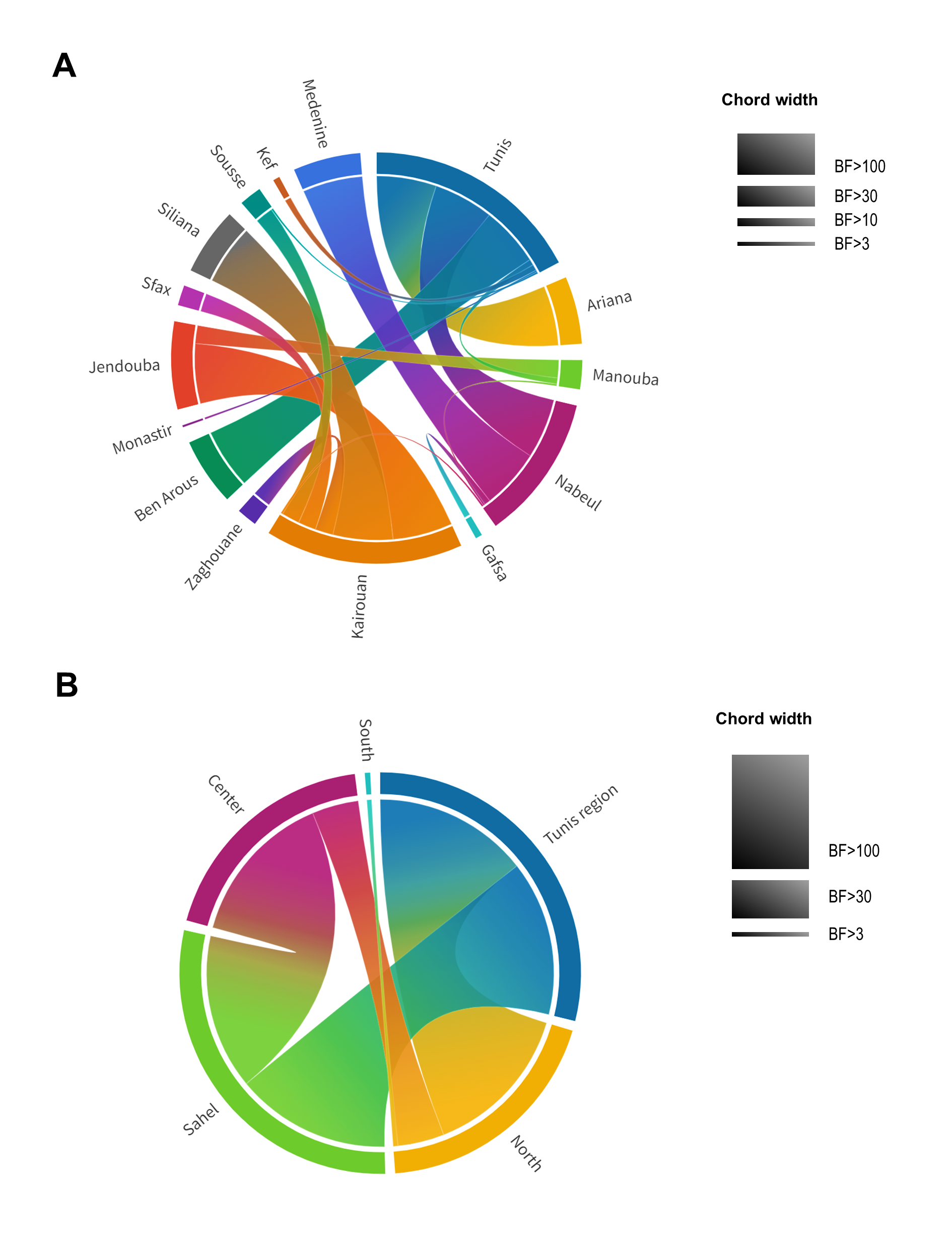

Supplement: Supplementary file 8 [file Image_1.TIF]
